# Effects of X-chromosome Tenomodulin Genetic Variants on Obesity in a Children’s Cohort and Implications of the Gene in Adipocyte Metabolism

**DOI:** 10.1038/s41598-019-40482-0

**Published:** 2019-03-08

**Authors:** Francisco Javier Ruiz-Ojeda, Augusto Anguita-Ruiz, Azahara I. Rupérez, Carolina Gomez-Llorente, Josune Olza, Rocío Vázquez-Cobela, Mercedes Gil-Campos, Gloria Bueno, Rosaura Leis, Ramón Cañete, Luis A. Moreno, Angel Gil, Concepcion Maria Aguilera

**Affiliations:** 1 Department of Biochemistry and Molecular Biology II, Institute of Nutrition and Food Technology “José Mataix”, Center of Biomedical Research, University of Granada, Avda. del Conocimiento s/n., 18016 Armilla Granada, Spain; 2 Instituto de Investigación Biosanitaria IBS.GRANADA, Complejo Hospitalario Universitario de Granada, Granada, 18014 Spain; 3 Spanish Biomedical Research Centre in Physiopathology of Obesity and Nutrition (CIBERobn), Instituto de Salud Carlos III (ISCIII), Madrid, 28029 Spain; 4 Unit of Investigation in Nutrition, Growth and Human Development of Galicia, Pediatric Department (USC), Instituto de Investigación Sanitaria de Santiago de Compostela (IDIS), Complexo Hospitalario Universitario de Santiago, Santiago de Compostela, Spain; 5 Growth, Exercise, NUtrition and Development (GENUD) Research Group, Universidad de Zaragoza, Zaragoza, Spain; 6 Instituto Agroalimentario de Aragón (IA2), Instituto de Investigación Sanitaria de Aragón (IIS Aragón),, Zaragoza, Spain; 7 Department of Paediatrics, Reina Sofia University Hospital, Institute Maimónides of Biomedicine Investigation of Córdoba (IMIBIC), University of Córdoba, Avda Menéndez Pidal s/n, 14004 Córdoba, Spain

**Keywords:** Genomics, Molecular biology

## Abstract

Tenomodulin (TNMD) is a type II transmembrane glycoprotein that has been recently linked to obesity, and it is highly expressed in obese adipose tissue. Several sex-dependent associations have been observed between single-nucleotide polymorphisms (SNPs) of the *TNMD* gene, which is located in the X-chromosome, and obesity, type 2 diabetes mellitus (T2DM), and metabolic syndrome in adults. On the other hand, results are lacking for children. We aimed (i) to study the association between *TNMD* genetic variants and metabolic complications related to childhood obesity and (ii) to investigate the function of TNMD in human adipocytes. We conducted a case-control, multicenter study in 915 Spanish children and demonstrated significant positive associations between *TNMD* genetic variants and BMI z-score, waist circumference, fasting glucose, and insulin resistance in boys, highlighting the SNP rs4828038. Additionally, we showed a BMI-adjusted inverse association with waist circumference in girls. Second, *in vitro* experiments revealed that TNMD is involved in adipogenesis, along with glucose and lipid metabolism in differentiated adipocytes, and these effects may be mediated through AMPK activation. Hence, these results suggest that *TNMD* genetic variants could be potentially useful as early life risk indicators for obesity and T2DM. In addition, we support the fact that TNMD exhibits significant metabolic functions in adipocytes.

## Introduction

Childhood obesity is a major health problem (GBD 2015 Obesity Collaborators) characterized by an expansion of the adipose tissue (AT)^1^. Many children who are overweight or suffer from obesity before puberty maintain obesity in early adulthood, which is associated with increased morbidity and mortality^2^. The expansion of AT implies metabolic alterations that are mainly related to glucose and lipid metabolism^3^. White adipose tissue (WAT) is the main site for energy storage, but it is also an endocrine organ that secretes cytokines and adipokines^4^. White subcutaneous adipose tissue (SAT) and white visceral fat depots (VAT) represent 80% and 20% of total body fat storage, respectively. VAT size is strongly associated with insulin resistance, and it is well established that VAT and SAT are different with respect to adipocyte size and metabolic activity^5^.

Tenomodulin (*TNMD*) was identified as a novel gene in 2001 by Brandau *et al*.^6^ and Shukunami *et al*.^7^, and it is located in the human Xq22 region, where it spans approximately 15 kb^6,7^. TNMD is a type II transmembrane protein; it is described as an angiogenesis inhibitor and is highly expressed in hypovascular connective tissues such as tendons and cartilage^6,8^. Indeed, TNMD contains a putative proteinase cleavage and two glycosylation sites where the C-terminus of the protein is cleaved in those tissues^9–12^. Furthermore, its expression in human AT has been recently observed to be higher in obesity and lower after diet-induced weight loss^13^. Analyses of AT *TNMD* expression in obese and lean subjects have also shown that *TNMD* mRNA is correlated with body mass index (BMI) in adults^14–16^. In line with these results, our research group previously found that *TNMD* was five-fold upregulated in the VAT of prepubertal children with obesity, compared with their normal-weight counterparts^17^. Furthermore, TNMD is known to promote human adipocyte differentiation and to act as a protective factor against insulin resistance in obese VAT^18^.

Likewise, several studies have indicated that single-nucleotide polymorphisms (SNPs) in the *TNMD* gene are associated with BMI, serum low-density lipoprotein cholesterol (LDL-c) levels, and inflammatory factors in adults in a sex-specific manner^19^. Specifically, the SNPs rs2073162 and rs2073163 have been associated with type 2 diabetes mellitus (T2DM) in men, central obesity in women and inflammation in men and women^19–23^. On the other hand, results are lacking for children.

At the GWAS level, none of the analyses that have been conducted on obesity traits have reported associations for *TNMD* SNPs. Since the X-chromosome has often been less scrutinized because of the unique statistical challenges it presents^24,25^, the X-chromosomal location of *TNMD* could be one of the reasons why its genetic variants have not been widely studied in the genetic context of obesity. Despite this, the X-chromosome has been proposed as a potential source of missing heritability and an important genomic region to be included into analyses^26^. Considering all this and the availability of new tools to overcome these complexities^25,27–30^, the present work was undertaken to study the effects of *TNMD* genetic variants in children with obesity and to evaluate the potential metabolic function of this gene in human adipocytes. First, we studied the association between *TNMD* genetic variants and metabolic complications related to childhood obesity. Second, through gene silencing, we aimed to demonstrate that TNMD is required for adipocyte metabolism in fully differentiated adipocytes. To the best of our knowledge, this is the first study to report an association between *TNMD* SNPs and childhood obesity while supporting the implication of *TNMD* in adipocyte metabolism.

## Results

### *TNMD* genetic variants are associated with BMI z-score in boys

**Figure 1:**
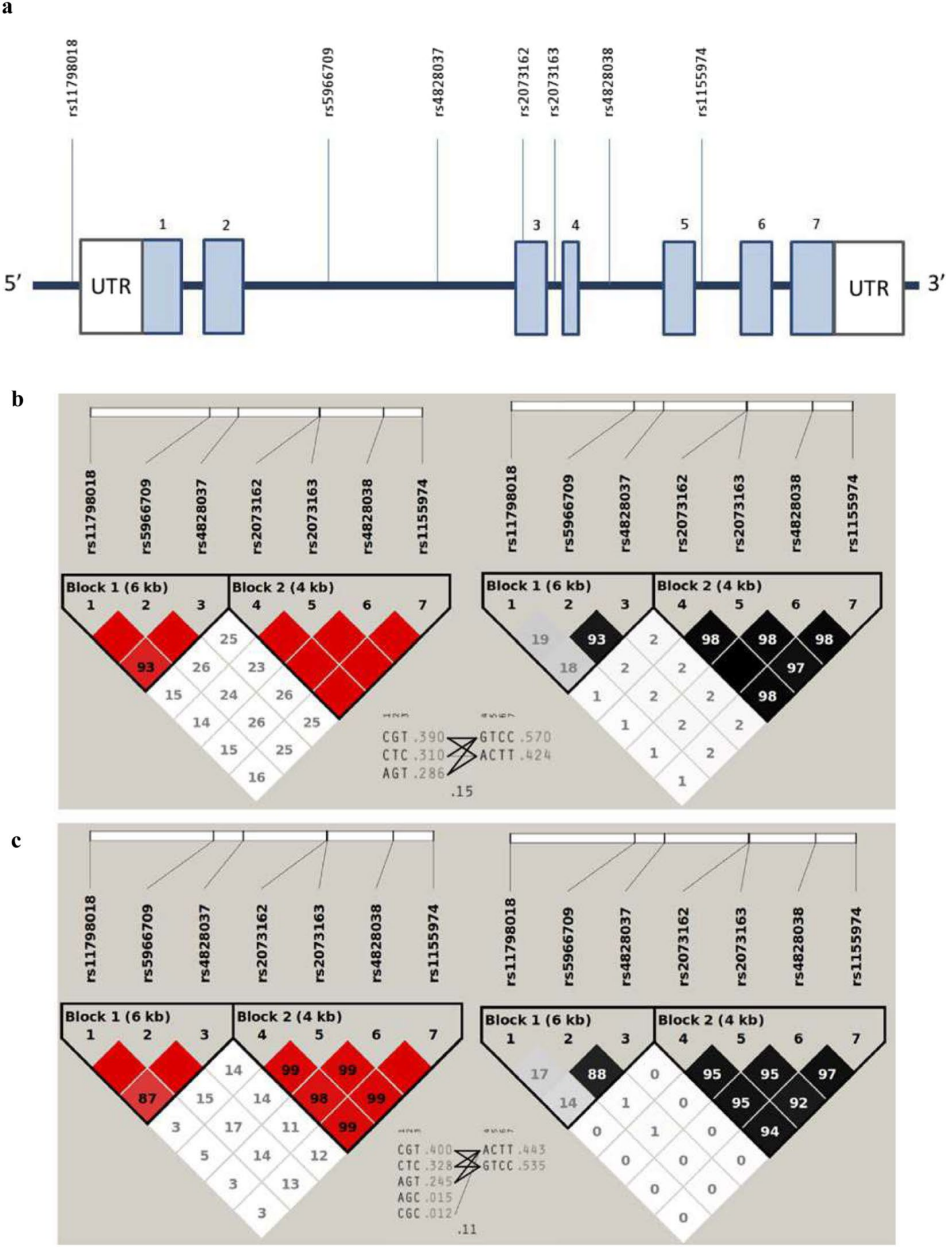
Location of selected markers in the *TNMD* gene and linkage disequilibrium (LD) analyses. (**a**) Light blue boxes represent exons, while the connecting blue lines are introns. Abbreviations: rs, reference SNP code; UTR, untranslated region. (**b**,**c**) show the LD pattern of the region in boys and girls, respectively. Left red triangles represent D′ values while right the black/gray triangles indicate R^2^ values. Triangle frames indicate observed haploblocks according to the solid spine of LD; the first consists of rs11798018, rs5966709, and rs4828037, and the second consists of rs2073162, rs2073163, rs4828038, and rs1155974. Between triangles, we listed each haplotype in a block along with its population frequency and connections from one block to the next. In the crossing areas, a value of multiallelic D′ is shown. This represents the level of recombination between the two haploblocks.

The anthropometric, clinical, and metabolic characteristics of the children participating in the present study are shown in the supplementary material according to obesity status (Supplementary Table S1). Minor allele frequencies (MAFs) of all markers studied are listed in Table 1. All SNPs showed MAFs above 5% regardless of the obesity class. Given the location of *TNMD* in a sex chromosome, all genetic analyses were conducted separately for boys and girls. The linkage disequilibrium (LD) pattern of the region of *TNMD* that was studied is presented in Fig. 1; two previous literature-reported blocks were also identified in our population in a sex-stratified manner: haploblock-1 (rs11798018, rs5966709, and rs4828037) and haploblock-2 (rs2073162, rs2073163, rs4828038, and rs1155974)^20^. All SNPs within the haploblock-2 showed significant and positive association with the BMI z-score in boys but not in girls (Table 1). Conversely, no association was identified between variants of the haploblock-1 and BMI z-score in any sex group. Among the associated SNPs within the haploblock-2, the rs2073162 and the rs4828038 exhibited the highest effect sizes and the most significant P values. All mentioned associations remained statistically significant after applying multiple-test correction by False Discovery Rate (FDR). Instead, only the rs2073162 association stood multiple-test correction by *Bonferroni* adjustment (Table 1).

**Table 1:** Association between *TNMD* SNPs and BMI z-score in children.

SNP	MAF					*P*-value	*FDR adjusted P*-value	*BONF adjusted P*-value
	A1/	Normal-weight	Overweight	Obese	β (95%CI)			
	A2							
**rs11798018**	A/C							
Females		0.272	0.269	0.263	0.11 (−0.13, 0.36)	0.369	0.4696	1
Males		0.283	0.242	0.280	0.14 (−0.34, 0.63)	0.561	0.6545	1
**rs5966709**	T/G							
Females		0.289	0.294	0.355	0.14 (−0.07, 0.35)	0.202	0.404	1
Males		0.325	0.286	0.316	0.07 (−0.39, 0.52)	0.762	0.8206	1
**rs4828037**	C/T							
Females		0.297	0.332	0.379	0.15 (−0.06, 0.36)	0.161	0.404	1
Males		0.350	0.281	0.332	0.01 (−0.43, 0.46)	0.952	0.952	1
**rs2073162**	A/G							
Females		0.447	0.468	0.463	0.11 (−0.08, 0.30)	0.267	0.4262	1
Males		0.395	0.328	0.469	**0**.**65 (0**.**22**, **1**.**07)**	**0**.**003**	**0**.**0233**	**0**.**042**
**rs2073163**	C/T							
Females		0.445	0.479	0.476	0.11 (−0.09, 0.30)	0.274	0.4262	1
Males		0.402	0.345	0.471	**0**.**59 (0**.**15**, **1**.**03)**	**0**.**008**	**0**.**028**	0.110
**rs4828038**	T/C							
Females		0.447	0.459	0.463	0.12 (−0.06, 0.31)	0.193	0.404	1
Males		0.395	0.311	0.465	**0**.**64 (0**.**21**, **1**.**06)**	**0**.**004**	**0**.**0233**	0.056
**rs1155974**	T/C							
Females		0.447	0.454	0.443	0.09 (−0.10, 0.29)	0.339	0.4696	1
Males		0.400	0.311	0.466	**0**.**62 (0**.**19**, **1**.**04)**	**0**.**005**	**0**.**0233**	0.070

BMI, body mass index; A1, minor allele; A2 major allele; MAF, minor allele frequency; β, Beta obtained under an additive model; CI, confidence interval. Linear regression analyses stratified by sex were performed under an additive model assuming TNMD locus escapes from the X-chromosome inactivation process. That is, while the female genotypes were coded 0, 1, or 2 according to 0, 1, or 2 TNMD SNP alleles, the genotypes for males were coded 0 or 1 according to 0 or 1 alleles.

No effects were reported for the haploblock-1 variants on any of the studied phenotypes, and rs4828038 was identified as a tag SNP within the haploblock-2 according to the Bakker’s method^31^; therefore, the paper will focus on all associations and findings that have been reported for this marker. A conditional joint multiple-SNP analysis for BMI z-score in boys further revealed there are no independent effects on the phenotype between all linked markers of the haploblock-2 (Supplementary Fig. S1). On this matter, our tag SNP rs4828038 might be a good representative marker for the region. Additionally, haplotype-based tests were performed to determine whether the reported associations remained statistically significant when each *TNMD* haploblock was analyzed as an allelic phase and not as independent single variants (Supplementary Table S2). As expected, the association between the haploblock-2 and the BMI z-score in boys remained statistically significant, even after applying a multiple-test correction.

### The tag SNP rs4828038 is associated with central adiposity and impaired glucose metabolism in boys, while it correlates with lower waist circumference in girls

To further explore the implication of *TNMD* in obesity and metabolic alterations, we studied the association between *TNMD* genetic variants and a range of additional anthropometric measurements and metabolic features including cardiovascular disease (CVD) and inflammation biomarkers (Table 2 and Supplementary Table S3).

**Table 2:** Association between rs4828038 *TNMD* and anthropometric, biochemical, and inflammation characteristics (mean (SD)) in children.

Phenotype	Genotypes						Β (95%CI)	*P*	*FDR adjusted P*	Β_BMI_ (95%CI)	*P*_*BMI*_	*FDR adjusted P*_*BMI*_
	T/T	n_T/T_	T/C	n_T/C_	C/C	n_C/C_						
**Waist circumference (cm)**												
Females	75.68 (15.11)	124	77.13 (16.68)	168	77.15 (15.13)	168	−0.42 (−2.05, 1.04)	0.611^a^	0.694	**−1**.**22 (−2**.**06**, **−0**.**38)**	**0**.**005**^**a**^	**0**.**024**
Males	80.9 (17.4)	157	NA	0	77.04 (17.3)	220	**3**.**76 (0**.**55**, **6**.**98)**	**0**.**022**^**a**^	0.088	−0.1 (−1.54, 1.35)	0.897^a^	0.962
**Waist to Height Ratio**												
Females	0.526 (0.09)	109	0.54 (0.10)	151	0.53 (0.09)	142	−0.002 (−0.01, 0.009)	0.701^a^	0.876	**−0**.**01 (−0**.**01**, **0**.**001)**	**0**.**030**^a^	0.172
Males	0.54 (0.10)	130	NA	0	0.53 (0.10)	194	0.015 (−0.007, 0.04)	0.183^a^	0.735	−0.002 (−0.01, 0.01)	0.678^a^	0.753
**Systolic BP (mmHg)**												
Females	104.8 (14.43)	108	106.9 (12.73)	148	104.9 (13.81)	136	0.006 (−1.65, 1.66)	0.995^b^	0.995	−0.46 (−1.95, 1.03)	0.542^b^	0.995
Males	107.7 (15.06)	131	NA	0	105.9 (16.31)	193	1.01 (−2.15, 4.17)	0.531^b^	0.995	0.07 (−2.91, 3.05)	0.963^b^	0.995
**Diastolic BP (mm Hg)**												
Females	62.82 (11.99)	108	65.17 (10.46)	148	65.46 (10.77)	136	−1.3 (−2.68. 0.09)	0.067^b^	0.332	**−1**.**60 (−2**.**91**, **−0**.**30)**	**0**.**016**^**b**^	0.102
Males	65.55 (11.31)	131	NA	0	63.82 (10.49)	193	1.53 (−0.82, 3.88)	0.204^b^	0.446	0.96 (−1.32, 3.23)	0.410^b^	0.708
**Glucose (mg/dl)**												
Females	84.7 (7.93)	132	83.64 (6.89)	170	84.37 (7.02)	172	0.16 (−0.66, 0.97)	0.707^a^	0.980	0.18 (−0.62, 0.99)	0.658^a^	0.906
Males	85.9 (8.45)	171	NA	0	83.71 (8.43)	235	**2**.**22 (0**.**57**, **3**.**87)**	**0**.**009**^**a**^	**0**.**049**	**2**.**21 (0**.**55**, **3**.**88)**	**0**.**009**^**a**^	0.054
**Insulin (mU/dl)**												
Females	12.85 (10.15)	131	13.5 (9.54)	164	11.63 (7.10)	167	0.81 (−0.14, 1.76)	0.094^a^	0.196	0.72 (−0.15, 1.59)	0.104^a^	0.443
Males	10.86 (7.67)	166	NA	0	9.50 (7.56)	224	1.39 (−0.02, 2.79)	0.053^a^	0.196	0.74 (−0.55, 2.02)	0.261^a^	0.443
**HOMA-IR**												
Females	2.76 (2.36)	131	2.82 (2.04)	163	2.44 (1.55)	166	0.2 (−0.01, 0.41)	0.064^a^	0.186	0.18 (−0.02, 0.38)	0.075^a^	0.386
Males	2.34 (1.79)	165	NA	0	2.02 (1.72)	224	**0**.**33 (0**.**009**, **0**.**66)**	**0**.**044**^**a**^	0.186	0.19 (−0.11, 0.50)	0.207^a^	0.386
**QUICKI**												
Females	0.35 (0.05)	131	0.34 (0.04)	163	0.35 (0.04)	165	0.0001 (−0.004, 0.004)	0.953^a^	0.952	0.0005 (−0.003, 0.004)	0.787^a^	0.810
Males	0.35 (0.04)	165	NA	0	0.37 (0.05)	224	**−0**.**01 (−0**.**02**, **−0**.**003)**	**0**.**006**^**a**^	**0**.**038**	**−0**.**008 (−0**.**02**, **−0**.**0004)**	**0**.**039**^**a**^	0.232
**TAG (mg/dl)**												
Females	72.57 (36.18)	132	71.58 (32.59)	170	67.1 (27.12)	172	2.86 (−0.74, 6.46)	0.120^a^	0.615	2.63 (−0.87, 6.13)	0.142^a^	0.878
Males	65.53 (35.37)	172	NA	0	64.32 (35.51)	234	1.35 (−5.59, 8.29)	0.703^a^	0.915	−1.00 (−7.74, 5.73)	0.771^a^	0.905
**HDL-c (mg/dl)**												
Females	52.32 (14.03)	130	51.95 (13.77)	168	53.39 (13.69)	171	−0.6 (−2.17, 0.96)	0.451^a^	0.683	−0.35 (−1.76, 1.06)	0.625^a^	0.960
Males	55.25 (15.1)	169	NA	0	56.69 (16.53)	231	−1.53 (−4.67, 1.60)	0.338^a^	0.683	0.20 (−2.64, 3.04)	0.892^a^	0.960
**IL-6 (ng/l)**												
Females	3.86 (7.22)	106	2.78 (4.51)	152	2.21 (3.83)	151	**0**.**80 (0**.**17**, **1**.**44)**	**0**.**014**^**a**^	**0**.**047**	**0**.**75 (0**.**12**, **1**.**38)**	**0**.**021**^**a**^	0.073
Males	3.25 (6.29)	142	NA	0	3.53 (5.80)	205	−0.30 (−1.59, 0.98)	0.642^a^	0.818	−0.38 (−1.68, 0.92)	0.568^a^	0.727

BMI, body mass index; BP, blood pressure; HOMA-IR, homeostasis model assessment for insulin resistance; QUICKI, quantitative insulin sensitivity check index; TAG, triglycerides; HDL-C, high-density lipoprotein cholesterol; IL, interleukin; MAF, minor allele frequency; β_BMI_, Beta obtained under an additive model adjusted for BMI; P-value_BMI_, P (P value) obtained under an additive model adjusted for BMI; CI, confidence interval; NA, not applicable. Linear regression analyses stratified by sex were performed under an additive model assuming *TNMD* locus escapes from the X-chromosome inactivation process. That is, while the female genotypes were coded 0, 1, or 2 according to 0, 1, or 2 *TNMD* SNP alleles, the genotypes for males were coded 0 or 1 according to 0 or 1 alleles. ^a^Adjusted for age. ^b^Adjusted for age and height.

Concerning anthropometric indicators of central obesity, a statistically significant and risky correlation was observed between the rs4828038-T-allele and waist circumference (WC) in boys, which disappeared after adjusting the model for BMI confounding. This finding did not remain statistically significant after applying *FDR* multiple-test correction neither. Conversely, we identified a protective association between the rs4828038-T-allele and WC in girls. The finding remained statistically significant after adjusting the model for BMI confounding (Table 2). Interestingly, this result also stood multiple-test correction and showed a 2.4% FDR value. Other central-adiposity indicators such as the waist-to-height ratio (WHR) reported equal findings in girls but did not reach multiple-test significance.

Regarding glucose metabolism, the rs4828038-T-allele was associated with higher levels of fasting glucose and higher values of homeostatic model assessment for insulin resistance (HOMA-IR) in boys. In the same way, the rs4828038-T-allele was also associated with a lower quantitative insulin sensitivity check index (QUICKI) (Table 2). All results were obtained under an additive model adjusted for age. Specifically, the correlation between our tag SNP and fasting glucose levels reached both nominal and multiple-test significance, exhibiting an FDR value of 4.9%. Significant results were also obtained after adjusting the model for BMI confounding but with only nominal statistical significance (P = 0.009). In relation to HOMA-IR, each rs4828038-T-allele copy increased the index value by 0.33 units in comparison to the rs4828038-C-allele. For the QUICKI index, we reported a risky and statistically significant correlation in boys that stood multiple-test FDR significance. The same results were obtained after adjusting the model for BMI confounding but with only nominal statistical significance (P = 0.039). No additional associations were reported regarding our tag SNP and any other glucose metabolism phenotype in neither boys nor girls.

According to previous studies relating *TNMD* SNPs to inflammatory traits and diseases such as age-related macular degeneration (AMD) or T2DM^21,32^, we investigated the association between the rs4828038 SNP and inflammation, CVD risk markers and adipokines (Table 2 and Supplementary Table S3). Interestingly, we reported a significant and positive correlation between the rs4828038-T-allele and interleukin (IL)-6 levels in girls, which remained significant after multiple-test correction (FDR = 4.7%). Concordant results were obtained after adjusting the model for BMI confounding but with only nominal significance (P = 0.021). Associations in line with this result have been previously identified in adult females in relation to *TNMD* haploblock-1 SNPs^21^, which suggests that *TNMD* could mediate its putative effects on obesity via low-grade inflammation. Nonsignificant results were observed for the rest of the analyzed traits of the block regardless of the sex.

Other metabolic features such as blood pressure and lipid traits were also investigated. We detected a significant association between the rs4828038-T-allele and diastolic blood pressure (DBP) in girls when adjusting the model for BMI confounding (Table 2). The result did not remain statistically significant after applying multiple-test correction, showing an FDR value of 10.2%. Remaining phenotypes of the block did not exhibit any significant correlation with analyzed markers (Table 2 and Supplementary Table S3).

### TNMD is associated with adipogenesis and lipid metabolism in human adipocytes

**Figure 2:**
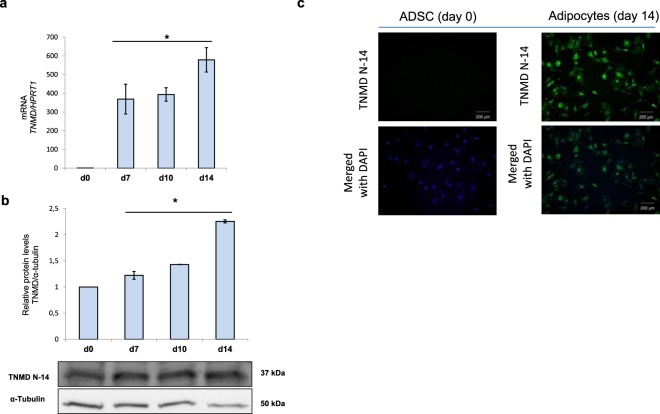
TNMD expression during adipogenic differentiation. (**a**) Gene expression of *TNMD* at various time points during adipogenic differentiation in human adipose-derived stem cells (ADSCs); mRNA levels were normalized to those of hypoxanthine-guanine phosphoribosyltransferase-1 (*HPRT1*) and presented as fold-change, as calculated using the Pfaffl method. (**b**) TNMD protein levels from cell lysates were analyzed by Western blotting using a specific antibody against TNMD (N-14), normalized to the internal control (α-tubulin), and expressed as fold-change; the lower section presents a representative crop blot. **(c)** Immunofluorescent staining of ADSCs (d0) and differentiated adipocytes at day 14 N-14 terminal domains of TNMD (green) and 4.6-diamidino-2-phenylindole (DAPI; blue; scale bar, 200 μm). All values are expressed as the means ± SEM of three independent experiments. Significant differences were identified using the nonparametric Mann-Whitney U test; *P-value < 0.05.

Based on the data regarding the relationship between *TNMD* genetic variants and the BMI z-score and, especially, WC, together with our previously published results that described a highly significant upregulation of *TNMD* expression in the VAT of children with obesity^17^, we performed a functional *in vitro* study. The aim of this study was to elucidate the role of *TNMD* in human adipocytes, as the major constituent cells in AT. To assess *TNMD* gene and protein expression in human adipocytes from adipose-derived stem cells (ADSCs), we determined the mRNA and protein levels at various times during adipogenic differentiation. In agreement with previous reports^13,18^, we found that *TNMD* expression and protein levels were significantly upregulated in human differentiated adipocytes at day 14 compared with ADSCs at day 0. However, we did not find any significant differences between days 7, 10, and 14. Immunofluorescence images also showed the differences between TNMD expression at days 0 and 14 (Fig. 2).

**Figure 3:**
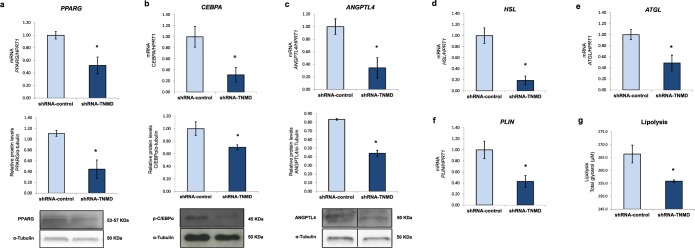
TNMD promotes adipogenesis and impairs lipid metabolism in human adipocytes. Human adipocytes were transfected with an adenovirus-5 containing a shRNA-TNMD and shRNA-control (scrambled) at day 14 of adipogenesis induction. **(a–c**) Peroxisome proliferator-activated receptor gamma (PPARG), CCAAT/enhancer-binding protein alpha (CEBPA) and angiopoietin-like 4 (ANGPTL4) mRNA and protein levels were determined in the shRNA-TNMD and shRNA-control adipocytes. Protein levels in cell lysates were analyzed by Western blotting using specific antibodies against PPARG, CEBPA and ANGPTL4, normalized to the internal control (α-tubulin), and expressed as fold-change; the lower section shows a representative crop blot. **(d**–**f)** Hormone-sensitive lipase (*HSL*) gene expression, adipose triglyceride lipase (*ATGL*), and perilipin (*PLIN*) gene expression were determined in the shRNA-TNMD and shRNA-control adipocytes. (**g**) Glycerol levels (µM) in cell supernatants after treatment with shRNA-TNMD. All values are expressed as the means ± SEM of three independent experiments. Significant differences were identified using the nonparametric Mann-Whitney U test; *P-value < 0.05.

In addition, it has been demonstrated that *TNMD* inhibition blocks adipogenesis in Simpson-Golabi-Behmel syndrome (SGBS) preadipocytes and benefits VAT expansion in mice. Since TNMD is required for adipocyte differentiation in SGBS adipocytes^18^, we first confirmed that TNMD was also required for human adipogenesis in ADSCs (Supplementary Fig. S2). Additionally, *TNMD* inhibition expression in fully differentiated adipocytes at day 14, downregulated the gene and protein expression of peroxisome proliferator-activated receptor gamma (PPARG), CCAAT/enhancer-binding protein alpha (CEBPA) and angiopoietin-like 4 (ANGPTL4) in TNMD-knocked-down adipocytes (Fig. 3).

Regarding lipid metabolism, knock-down of *TNMD* led to reduced lipolysis as observed by decreased extracellular glycerol levels in cell supernatants (Fig. 3g). In addition, the expression levels of lipases such as hormone-sensitive lipase (*HSL*), adipose triglyceride lipase (*ATGL*), and perilipin (*PLIN*) were significantly downregulated upon shRNA-TNMD treatment (Fig. 3d–f), as was ANGPTL4, which is a mediator of intracellular lipolysis in adipocytes (Fig. 3c).

Since TNMD is involved in the regulation of tenocyte proliferation, tendon development, and angiogenesis inhibition^12^, we investigated vascular endothelial growth factor A (VEGFA) gene and protein expression in shRNA-mediated knocked-down *TNMD* cells. However, we did not observe significant changes (Supplementary Fig. S3). In this sense, no differences in blood vessel morphology and density have been previously found in TNMD transgenic mice^18^.

### TNMD knock-down impairs glucose metabolism in human adipocytes

**Figure 4:**
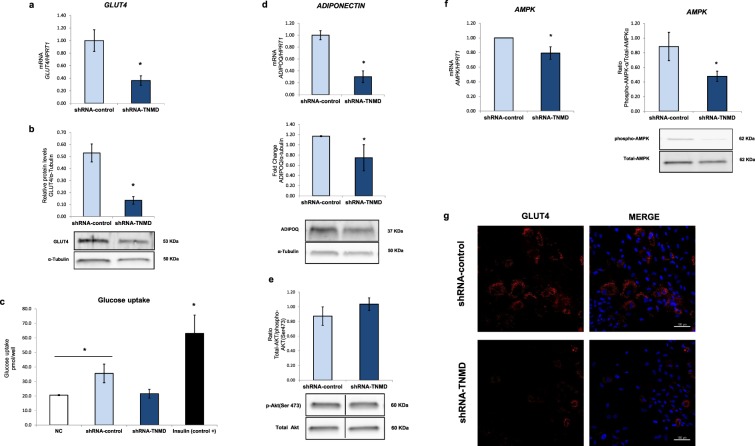
TNMD is involved in glucose metabolism in human differentiated adipocytes. Human adipocytes were transfected with an adenovirus-5 containing a shRNA-TNMD and shRNA-control (scrambled) at day 14 of adipogenesis induction. **(a)** Glucose transporter 4 (*GLUT4*) mRNA levels were normalized to those of hypoxanthine-guanine phosphoribosyltransferase-1 (*HPRT1*), and the data from three independent experiments are presented as the fold-change, which was calculated using the Pfaffl method. **(b)** GLUT4 protein levels from cell lysates were analyzed by Western blot using a specific antibody against GLUT4, normalized to the internal control (α-tubulin) and expressed as fold-change; the lower section shows a representative crop blot. **(c)** Glucose uptake levels in shRNA-TNMD-treated adipocytes compared with the shRNA-control or insulin (1 μM, 30 min) as a positive control. **(d)** Adiponectin (*ADIPOQ*) mRNA and protein levels expressed as fold-change. **(e)** Ratio phosphor-AMPKα/total-AMPKα. **(f)** Ratio phosphor-AKT/total-AKT. **(g)** Immunofluorescent staining of adipocytes at day 14 with GLUT4 (red) and 4.6-diamidino-2-phenylindole (DAPI; blue; scale bar, 200 μm) in the shRNA-control and shRNA-TNMD-treated adipocytes. All values are expressed as the means ± SEM of three independent experiments. Significant differences were identified using the nonparametric Mann-Whitney U test; *P-value < 0.05.

To test whether TNMD plays a role in glucose metabolism in adipocytes, *TNMD* was inhibited in fully differentiated human adipocytes at day 14. In this experiment, we observed that glucose transporter 4 (*GLUT4*) gene and protein expression were downregulated when *TNMD* expression was inhibited (Fig. 4a,b). This finding was supported by immunofluorescence images that showed reduced GLUT4 protein levels in shRNA-*TNMD*-treated adipocytes (Fig. 4g). However, *TNMD* inhibition did not affect basal glucose uptake in a significant manner; however, there was a tendency toward a reduction by approximately 1.5-fold (*P*-value: 0.08) (Fig. 4c). Adiponectin mRNA and protein levels were also decreased in *TNMD*-knocked-down adipocytes (Fig. 4d).

Regarding the activation of kinases involved in glucose metabolism, we observed a lower activation of AMP-activated protein kinase-alpha (AMPKα) and no differences in AKT (phosphor-AKT, Ser473) in *TNMD*-inhibited adipocytes. Thus, the association between TNMD and glucose metabolism in human adipocytes could be mediated by AMPK.

### TNMD knock-down triggers inflammation in human adipocytes

**Figure 5:**
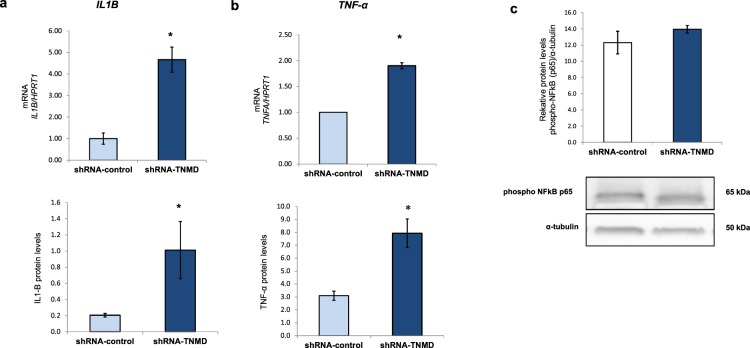
TNMD triggers inflammation in human differentiated adipocytes. (**a**) mRNA expression and protein levels of interleukin 1-β (*IL-1B*); **(b**) mRNA expression and protein levels of tumor necrosis factor-α (*TNF-α*), and mRNA levels were normalized to those of hypoxanthine-guanine phosphoribosyltransferase-1 (*HPRT1*); TNF-α and IL1B protein levels were analyzed by XMap technology (Luminex) as indicated in the methods section. **(c)** Phospho-NFκB p65 protein levels were analyzed by Western blot using a specific antibody against phospho-NFκB p65, normalized to the internal control (α-tubulin), and expressed as the fold-change. The lower section shows a representative crop blot. The data from three independent experiments are presented as the means ± SEM. Significant differences were identified using the Mann-Whitney U test; *P-value < 0.05.

Many studies have reported that inflammation occurs in adipocytes associated with obesity, which is further related to metabolic dysfunction and insulin resistance^33,34^. Thus, we next studied the inflammatory status of *TNMD*-inhibited human differentiated adipocytes. In our study, *TNMD*-knocked-down cells showed an upregulation of inflammatory markers such as *IL1-β* and tumor necrosis factor-α (*TNF-α*) mRNA. Furthermore, we observed a significant upregulation of the protein levels in the shRNA-TNMD-treated adipocytes compared with that in cells transfected with shRNA-control (*P*-value < 0.05) (Fig. 5). However, we did not observe significant differences in the activation of the nuclear factor kappa-light-chain-enhancer of activated B cells (NF-κB) pathway through p65 subunit phosphorylation. Although TNMD could play a role against inflammation in adipocytes, more studies are needed to elucidate the underlying mechanism.

## Discussion

In the present study, we show that X-chromosome *TNMD* genetic variants are associated with childhood obesity and metabolic alterations in a cohort of Spanish children. Particularly, we show that the tag SNP rs4828038 is associated with anthropometry, glucose metabolism alterations, and increased levels of pro-inflammatory biomarkers in a sex-specific manner. Furthermore, by *in vitro* gene silencing, we demonstrate that TNMD is required for an adequate glucose and lipid metabolism, and it plays a role in the control of inflammation in cultured human adipocytes.

Genetic association studies are commonly focused on autosomal variants, and genetic polymorphisms in sex chromosomes are often neglected^25^. Notwithstanding, the study of sex chromosomes might help to clarify the role of several genes in the development of many diseases, especially complex human traits that exhibit gender disparity in risk or symptoms^28,35,36^. Studies that employ mouse models and allow the distinction of gonadal from chromosomal effects have revealed that X-chromosome dosage influences food intake, which in turn affects adiposity and the occurrence of adverse metabolic conditions such as hyperinsulinemia, hyperlipidemia, and fatty liver^37^.

To date, the present study is the first to analyze and detect associations between *TNMD* X-chromosome genetic variants and obesity and its metabolic complications in a cohort of children. For that purpose, we quantified our power in 96.12% to detect small GWAS-size genetic effects (estimated in F^2^ = 0.02) at an alpha level of 0.05.

Specifically, we found a risky correlation between the rs4828038, tag SNP of the *TNMD* haploblock-2, and the BMI z-score and WC in boys. Interestingly, findings related to BMI z-score remained statistically significant after applying multiple-test correction. Regarding these anthropometric measurements, others authors such as Tolppanen *et al*.^20^, have also reported *TNMD* associations^20^. Surprisingly, they have found a protective association between variants located in the haploblock-1 (rs11798018, rs5966709 and rs4828037) and BMI and weight in adult European men. Although these findings are reported for a different haploblock than our associated haploblock-2 SNP, such controversy merits special attention. A possible explanation might rely on the fact that both haploblock-1 and haploblock-2 could elicit contrasting effects on *TNMD* expression through, for example, the alteration of microRNA targets sites or the generation of different splicing patterns. Other sources of variability might also rely on the fact that we are studying a cohort of children, while Tolppanen *et al*. focus on adult population with an advanced status of impaired glucose tolerance (IGT) and T2DM. In this regard, further *TNMD* functional genetic studies are needed to clarify such an issue.

Regarding girls, we showed a protective correlation between our tag SNP rs4828038 and WC and WHR. In both associations, BMI was controlled for as a confounder. Specifically, the association between our tag SNP and WC further stood multiple-test correction (FDR = 2.4%). Interestingly, our findings are in accordance with prior works of Tolppanen *et al*.^20^, who detected an association between the rs2073162-A-allele (marker in complete LD with our tag SNP) and smaller horizontal diameters in adult females when adjusting the model by BMI^20^.

Considering all this, we can see how the same region (haploblock-2) appears as a risk factor for obesity in boys, while at the same time, it acts as a protective element for central obesity parameters in girls. Such sex-specific behavior in our study reflects the typical sexual dimorphism of the X-chromosome very well, and it could arise from some X-chromosome particularities including differential gene dosage, the escape from the X-chromosome inactivation (XCI) and the existence of distinct genomic imprint mechanisms^37–41^. To account for all these X-chromosome particularities, several specific steps and procedures have been implemented following published recommendations^25,27–30^ (see the method section).

Regarding glucose metabolism, several risky correlations were found for the rs4828038 SNP in boys. Particularly, we identified that the rs4828038-T-allele was associated with higher levels of fasting glucose and HOMA-IR, as well as lower values of QUICKI index. Interestingly, QUICKI and glucose associations remained statistically significant also after controlling for BMI confounding. In analyses without BMI-confounding adjustment, QUICKI and glucose insights further reached *FDR* multiple-test significance. Altogether, these associations are in concordance with previous findings that have been obtained by Tolppanen *et al*.^20^ during a 3-year follow-up study. For two *TNMD* SNPs (the rs2073163 and the rs1155974) in strong LD with our tag SNP, they showed that men carrying the C and T alleles (respectively) presented an altered oral glucose tolerance test in comparison to individuals with the T and C alleles. These markers, along with the rs2073162, were also associated with an increased risk to develop T2DM during a 5-year follow-up study conducted in men^20^. Considering all this, we could hypothesize that the small alterations detected in the glucose metabolism of our boys according to the *TNMD* genotypes may be a premature signal of future complications during adulthood such as IGT or even its progression to T2DM. On this matter, *TNMD* genetic variants could be potentially useful as early life risk indicators for T2DM in male subjects. For girls we did not observe significant results in any glucose metabolism outcome. Previous studies in adults have reported contradictory findings in this regard.

In summary, although some of our genetic observations show a multiple-test level of statistical significance, it is important to stress, however, that they have not been corrected for between-trait multiple-test error. Thus, showed FDR corrected values are not study-wide robust and should be interpreted with caution. On the other hand, although we have taken some steps to account for main X-chromosome particularities, not all available suggestions were possible to incorporate in our study since this is a candidate-gene analysis instead of a GWAS approach. This, along with the fact that previous *TNMD* studies are statistically weak and barely accounted for X-chromosome specifications^20–22^, indicates that our study should be viewed as hypothesis-generating instead of a replication approach. On this matter, more detailed characterization in bigger and independent children samples as well as additional follow-up studies during adulthood are needed.

According to these results in children, *TNMD* SNPs are associated with impaired glucose metabolism and we previously found *TNMD* overexpression in VAT from prepubertal obese children^17^. Other studies have also described that *TNMD* expression is highly upregulated in human AT, increased in obesity^14–16^ and downregulated after diet-induced weight loss^13^ and that *TNMD* expression is predominant in adipocytes compared with stromal vascular fraction (SVF) cells^18^. Furthermore, *TNMD* expression promotes preadipocyte proliferation and adipogenesis in SGBS adipocytes, and they improved insulin sensitivity in *Tnmd* transgenic mice, which suggesting the protective role of TNMD in VAT to alleviate insulin resistance in obesity^18^. Consistent with these results, *TNMD* knock-down led to lower gene expression and protein levels of important transcription factors that are involved in adipogenesis such as *PPAR-γ* and *C/EBP-α*. On the other hand, as *TNMD* is expressed in dense connective tissues as tendons and ligaments, and the C-terminal domain could be processed as a soluble factor, this fraction could reach the adipose tissue and promote the adipogenic differentiation *in vivo*. However, further studies are needed to clarify this possible effect. Therefore, our results confirm the fact that TNMD promotes adipogenic differentiation, and it could be implicated as a protective factor that contributes to AT expansion.

The reduced lipolysis observed in *TNMD*-knocked-down adipocytes could be explained by the reduced gene expression and protein levels of PPAR-γ, as well as by the reduced AMPK activation because AMPK is the master regulator of metabolism. Indeed, it has been described that *PPARG2*-knocked-out adipocytes exhibit reduced lipolysis^42^; this could be explained by the lower expression of HSL^43^ and ATGL^44^, since they are both transcriptional targets of PPAR-γ. On the other hand, it has been reported that ANGPTL4 promotes the expression of genes involved in lipolysis in adipocytes^45^, and since *ANGPTL4* gene and protein levels were significantly downregulated when *TNMD* was inhibited, the results indicate that TNMD could be directly associated with lipid metabolism through ANGPTL4.

The association found between *TNMD* SNPs and fasting glucose levels in this study in children together with the higher 2-hour plasma glucose levels that was found in adults suggested a potential role for TNMD in adipocyte glucose metabolism. The confirmation of this hypothesis is another key finding in this study. We observed a down-regulation in gene expression and protein levels of the insulin-regulated glucose transporter GLUT4 in *TNMD*-knocked-down adipocytes and a tendency toward lower basal glucose uptake. Indeed, GLUT4 plays a critical role in the regulation of glucose metabolism and the maintenance of body glucose homeostasis^46^. Moreover, these results are in agreement with the observed lower adiponectin expression and lower AMPK activation. Adiponectin is the major secreted molecule of adipocytes and exerts multiple functions in regulation of energy homeostasis and glucose and lipid metabolism^47^. Adiponectin acts by increasing AMPK activity and stimulating GLUT4 expression^48^ and improves insulin sensitivity through inhibiting inflammatory signaling^49^. Upon activation, AMPK promotes GLUT4 expression and its translocation to the plasma membrane, thus favoring glucose uptake independent of insulin^50,51^.

On the other hand, it was reported that adiponectin knock-down did not affect the activation of AKT and p38MAPK (phosphorylation form/total form) but significantly decreased AMPK activation in insulin-responsive adipocytes^52^. In accordance with this finding, when *TNMD* expression was downregulated, we observed a reduced AMPK activation, lower adiponectin protein levels, and lower GLUT4 expression. However, we did not observe differences in AKT activation. These results suggest that the mechanism underlying the link between TNMD and glucose metabolism involves activation of AMPK. We also observed downregulation of C/EBP-α, which could directly bind and activate the GLUT4 gene promoter. It has been demonstrated that insulin and dexamethasone activate GLUT4 gene expression through C/EBP-α gene expression in brown adipose tissue^53^. Moreover, exogenous expression of C/EBP-α in C/EBP-null cells with PPAR-γ overexpression resulted in an increase in GLUT4 mRNA levels.

Obesity is also associated with an increased expression of pro-inflammatory mediators in AT, and this inflammation has been shown to interfere with glucose metabolism^54^. More specifically, TNF-α has been proposed as a link between adiposity and the development of insulin resistance, given its high expression in the AT of subjects with obesity^55,56^. TNF-α is mainly produced in adipocytes and induces tissue-specific inflammation and insulin resistance through a reduction in *GLUT4* expression^57–59^. Furthermore, TNF-α upregulates the expression of IL-6, IL1-β, and protein phosphatase 2C (PP2C), which, in turn, suppresses AMPK activity. In addition, TNF-α downregulates the expression of other important genes such as *adiponectin*, *C/EBP-α*, *PPAR-γ* and *PLIN*^60^. These circumstantial evidence support the fact that TNMD might be directly implicated in the protection against inflammation in the differentiated adipocytes since we found increased levels of TNF-α and IL1-β when *TNMD* was silenced. Additionally, IL-1β has been suggested to be involved in the development of insulin resistance^61^. Collectively, these data suggest that lowering the expression of *TNMD* in adipocytes leads to a pro-inflammatory status, which contributes to the dysregulation of glucose metabolism. Nevertheless, these results warrant further studies to elucidate the precise mechanisms.

Finally, in the present work, we find that *TNMD* is highly expressed in human ADSCs and that it is involved in their differentiation into mature adipocytes. This finding is in line with the study performed by Senol-Cosar *et al*.^18^, where *TNMD* was reported to be involved in human adipogenesis in preadipocytes^18^. However, depending on the cell line, the effects of TNMD on adipogenic differentiation are not completely clear. Shi *et al*.^62^ reported that *TNMD* overexpression did not affect the adipogenic differentiation in ASCs, which suggested that the endogenous *TNMD* gene is already expressed compared to other vascularized soft tissues^6^. Additionally, *TNMD* overexpression showed an inhibitory effect on the adipogenic differentiation of C3H10T1/2 and mMSC cells^63^. Interestingly, Lin *et al*.^61^ demonstrated the same results where *TNMD* knockout in mice exhibited significantly higher adipocyte accumulation, and *TNMD*^−/−^ TSPCs exhibited a higher rate of differentiation into adipocytes^61^. The diverse regulatory mechanism of TNMD is involved in different cell types, and further studies are needed to elucidate the specific TNMD function *in vivo*.

In conclusion, our data show that *TNMD* genetic variants, specifically rs4828038, which is a tag SNP within the presented haploblock 2, are associated with obesity and alterations in glucose metabolism in children. These results replicate previous findings that have been observed in adults and suggest that these markers could be potentially useful as early life risk factor indicators for obesity and the occurrence of alterations in glucose metabolism during adulthood. Additionally, we found a novel paradigm for TNMD in human adipocytes, which plays a role in adipogenesis and glucose and lipid metabolism, and report that these effects might be mediated through AMPK activation. Recent studies have indicated that TNMD is not only a glycoprotein that is expressed in the connective tissue with antiangiogenic properties but also beneficial for VAT expansion^18^. Thus, we demonstrated and supported the fact that TNMD presents significant metabolic functions in adipocytes and that it might be a potential therapeutic target to improve the glucose metabolic status.

## Methods

### Study population

In this case-control multicenter study, 915 Spanish children (438 boys and 477 girls) were included from three health institutions: Lozano Blesa University Clinical Hospital, Santiago de Compostela University Clinical Hospital, and Reina Sofia University Hospital. Childhood obesity status was defined according to the International Obesity Task Force (IOTF) reference for children^64^. There were 480 children in the obesity group, 177 in the overweight group, and 258 in the normal-BMI group. Inclusion criteria were European-Caucasian heritage and the absence of congenital metabolic diseases. The exclusion criteria were non-European Caucasian heritage; the presence of congenital metabolic diseases (e.g., diabetes or hyperlipidemia); under-nutrition; and the use of medication that alters blood pressure, glucose or lipid metabolism.

### Ethics statement

This study was conducted in accordance with the Declaration of Helsinki (Edinburgh 2000 revised), and it followed the recommendations of the Good Clinical Practice of the CEE (Document 111/3976/88 July 1990) and the legally enforced Spanish regulation, which regulates the clinical investigation of human beings (RD 223/04 about clinical trials). The Ethics Committee on Human Research of the University of Granada, the Ethics Committee of the Reina Sofía University Hospital of Cordoba, the Bioethics Committee of the University of Santiago de Compostela, and the Ethics Committee in Clinical Research of Aragon approved all experiments and procedures. All parents or guardians provided written informed consent, and the children gave their assent.

### Anthropometric and biochemical measurements

Body weight (kg), height (cm), and WC (cm) were measured using standardized procedures, and the BMI z-score was calculated based on the Spanish reference standards published by Sobradillo *et al*.^65^. Blood pressure was measured three times by the same examiner using a mercury sphygmomanometer and following international recommendations^66^. The biochemical analyses were performed at the participating university hospital laboratories following internationally accepted quality control protocols, including routine measures of lipid and glucose metabolism. QUICKI and HOMA-IR were calculated using fasting plasma glucose and insulin values. Adipokines, CVD risk, and pro-inflammatory biomarkers [adiponectin, leptin, resistin, TNF-α, IL-6, IL-8, total plasminogen activator inhibitor-1 (PAI-1), myeloperoxidase (MPO), matrix metalloproteinase-9 (MMP-9), soluble intercellular cell adhesion molecule-1 sICAM-1, and soluble vascular cell adhesion molecule-1 (sVCAM)] were analyzed on a Luminex 200 system (Luminex Corporation, Austin, Tex., USA) with human monoclonal antibodies (EMD MilliporeCorp, Billerica, MA) using MILLI*plex*^TM^ kits (HADK1MAG-61K, HADK2MAG-61K and HCVD2MAG-67K), as previously described^67^. High-sensitivity C-reactive protein (hsCRP) was determined using a particle-enhanced turbidimetric immunoassay (Dade Behring Inc., Deerfield, III, USA).

### Genotyping

Genomic DNA was extracted from peripheral white blood cells using two kits, the Qiamp® DNA Investigator Kit for coagulated samples and the Qiamp® DNA Mini & Blood Mini Kit for noncoagulated samples (QIAgen Systems, Inc., Valencia, CA, USA). All extractions were purified using a DNA Clean and Concentrator kit from Zymo Research (Zymo Research, Irvine, CA, USA).

Based on previously reported associations in adults^19–21^ and according to the *Tagger* program^31^, which was used to capture (at r^2^ = 0.8) common (MAF >= 5%) variants in European (CEU) HapMap population, we selected seven SNPs located at the *TNMD* locus for the present association analysis. The seven selected SNPs are distributed through all *TNMD* sequences and are representative of the region (Fig. 1a). Genotyping was performed by TaqMan allelic discrimination assay using the QuantStudio 12 K Flex Real-Time PCR System (Thermo Fisher Scientific, Waltham, MA, USA). The call rate exceeded 95% for all tested SNPs, except for rs11798018 (92.7%). Minor allele frequencies (MAF) of all SNPs were >2% and were similar to those reported for Iberian populations in Spain, in phase 3 of the 1000 Genomes Project. The Haploview software^68^ was used with specific sex chromosome settings to assess the LD between SNPs in a sex-stratified manner.

Given the number of markers, we considered several parallel approaches to correct for multiple hypothesis testing based on the number of SNPs^69^. Specifically, we employed correction based on the methods proposed by Holm (1979)^69^, Hommel (1988)^70^, Benjamini and Yekutieli (2001)^71,72^ and classical Bonferroni. To estimate the expected proportion of type I errors among the rejected hypotheses, we further computed false discovery rates (FDRs) as described in Benjamini and Hochberg^71^. Given the presence of linkage disequilibrium (LD), the FDR method is a proper approach that does not assume independence between markers.

### X-chromosome Inactivation (XCI) assumptions

XCI is one of the main X-chromosome particularities that affect the analytical process. Varieties of statistical tests are available for performing genetic analysis of the X-chromosome, and the choice will mainly depend on the XCI model assumed for each target-study gene.

After revising the literature, we found that the *TNMD* genetic region is barely covered by current studies and that there is a lack of XCI data in adipose-tissue-derived samples^73–75^. In this sense, a recent study^76^ reported that the XCI status of the *TNMD* region remains unknown. On the other hand, *TNMD* transcript levels have been reported to be two times higher in women than in men^14^.

Given this controversy and lack of evidence, both possibilities (‘escape’ and ‘XCI’) were tested in our work (see the method section ‘X-chromosome particularities and analyses’ for more details).

### X-chromosome particularities and analyses

Differential gene dosage between sexes, the escape from the XCI and the existence of distinct genomic-imprint mechanisms are some issues that make the X-chromosome a special region for genetic analyses. These particularities will determine important decisions that affect genotype calling, data imputation, quality control and statistics selection. The whole QC process was implemented in PLINK v1.07^77^.

Concerning genotype calling, algorithms that apply different procedures to male and female samples (e.g., Illuminus and CRLMM) have been proven to generally perform better than methods that do not (e.g., GenCall and GenoSNP)^78^. In our work, genotypes were called from fluorescence data files using the Applied Biosystems qPCR app module (ThermoFisher Cloud software) and the autocalling method. According to literature recommendations, sex information for each sample was supplied to the software and genotype calling was performed separately in both sexes. In this regard, although genotyped plates did not consist of only boys or girls, the balanced sex ratio of our population (477 f/438 m) (Supplementary Table S1**)** favored a better performance. Five signal clusters were identified (three in the case of females and two in the case of males). Next, sex information and scatter of the clusters were used to call the genotypes (AA, AB and BB for females, and A- and B- for males). Since the employed software also allows the option of using user-definable boundaries for data analysis, those samples classified as undetermined by the autocalling method were recalled using the manual option. A set of controls were used to deduce these questionable genotype calls. Outliers were omitted from the analysis.

Next, we checked *TNMD* SNPs for sex-specific allele frequencies, which can induce type I errors in some statistical analyses (especially in the case of unbalanced designs). Tested by means of the Fisher exact test, all SNPs showed nonsignificant P values and thus equal allele frequencies across sex groups (Supplementary Table S4). Two criteria concerning missing frequency were also employed (sex-specific missing frequencies and the differential missingness between sexes)^29,79^. As shown in Supplementary Table S5, our tag SNP passed the recommended filter in females (Missing Freq <= 2%) but not in males. Regarding the differential missingness test instead, only the rs11798018, rs4828037 and the rs2073163 passed the quality recommended filter (P ≥ 10^−7^). This test was performed in the PLINK software using the flag “*test-missing*” and replacing the phenotype column of the .*ped* file by sex information. Regarding additional MAF quality checks, all SNPs showed appropriated frequencies >1% by sex groups ( Supplementary Table S4). When analyzing the Hardy Weinberg equilibrium (HWE) in girls belonging to the normal-BMI group, all SNPs reported proper values (P ≥ 10^−4^) (Supplementary Table S6). According to this QC process, we ensured that there are no important genotyping errors and that our genetic data are reliable for further analyses.

Regarding high-level statistical analysis, both (‘escape’ and XCI) possibilities were tested as previously stated. At an initial phase, we assumed *TNMD* escapes from XCI and, thus, employed linear and logistic regressions (stratified by sex) under an additive model. That is, females were coded as 0, 1, or 2, according to the presence of 0, 1, or 2 risk alleles, while males were coded as 0 or 1 according to the presence of 0 or 1 risk allele. This codification was achieved in the PLINK software from the binary file using the flag “*–dosage*”. Additionally, we rerun all performed analyses using the X-chromosome specific version of common autosomal tests, developed by Clayton *et al*.^80^. Clayton’s test explicitly accounts for random XCI and allowed the inclusion of females and males together, thereby, increasing the statistical power. This secondary analysis was performed using the snpStats R package^81^. Major significant associations that were reported during the initial phase were further replicated using Clayton’s secondary approach (Supplementary Table S7).

### Data Records

The complete genetic data set in the present study complies with the requirements, and it has been uploaded into the European Genome-Phenome archive (EGA). With title “*X chromosomal genetic variants are associated with childhood obesity*”, the reference identifier of the project is EGAS00001002738 (2018). Data were uploaded according to obesity classes. The affected group (cases) was composed of children with obesity and those who were overweight (EGA EGAD00010001482 (2018)), and the control group was composed of normal-weight children (EGA EGAD00010001481 (2018)). Three by-experimental condition files are available online (.*bed*, .*bim* and .*fam* files). The .*bed* file contains the raw genotype data, while the .*bim* file describes the genotyped SNPs showing information related to chromosome number, SNP identifier, genetic distance in morgans (set as 0 for all markers), base-pair position (bp units) and allele letters. Finally, available *.fam* files contain information related to the study population (sample identifier, family and paternal identifiers (here set as 0), sex (1 for males and 2 for females) and phenotype group (1 for control and 2 for cases)). As previously stated, data are available online according to each experimental condition.

### Cell culture and adipogenic differentiation

Human adipose-derived stem cells (ADSCs) were purchased directly from Invitrogen (Gibco, Thermo Fisher Scientific, Carlsbad, CA, USA) (Gibco^TM^ Lot 2117, StemPro Human ADSCs). These commercially available ADSCs are isolated from normal (nondiabetic) women subcutaneous lipoaspirates that are collected during elective surgical liposuction procedures. ADSCs have been reported to differentiate into many different lineages, including chondrogenic, osteogenic, adipogenic, and neural lineages. We cultured, expanded, and differentiated ADSCs into adipocytes according to the manufacturer’s recommendations. Briefly, ADSCs were grown and expanded in appropriate sterile plastic dishes in complete Advanced-DMEM (Gibco, Thermo Fisher Scientific, Carlsbad, CA, USA) that was supplemented with 2 mM L-glutamine (25030, Gibco, Thermo Fisher Scientific, Carlsbad, CA, USA), 10% fetal bovine serum (FBS, PT-9000 H, Lonza, Basel, Switzerland), 100 U ml^−1^ penicillin and 100 µg ml^−1^ streptomycin (10378-016, Gibco, Thermo Fisher Scientific, Carlsbad, CA, USA). We incubated cells at 37 °C in a humidified atmosphere containing 5% CO_2_. The cell culture medium was replaced twice per week, and the cells were passaged up to a maximum of 6 times. To induce differentiation, we seeded cells in 35-mm dishes at a density of 30,000 cells/cm^2^, and we cultured them in MesenPRO RS^TM^ medium (12746-012, Gibco, Thermo Fisher Scientific, Carlsbad, CA, USA). At 90% confluency, the growth medium was replaced with StemPRO RS^TM^ adipogenic differentiation medium (A1007001, Gibco, Thermo Fisher Scientific, Carlsbad, CA, USA). ADSCs were incubated with differentiation medium for 14 days. We monitored and quantified adipogenesis through morphological examination of the cellular accumulation of lipid droplets via Oil Red O staining (234117, Sigma-Aldrich, St. Louis, MO, USA; Supplementary Fig. S4A) and by spectrophotometric determination of washed Oil Red O staining (Supplementary Fig. S4B). All treatments were performed on differentiated adipocytes at day 14.

### Adenoviral transduction

Briefly, knock-down of TNMD was performed simultaneously using four different shRNA (TR300905 from Santa Origene, Rockville, Maryland, USA) packed in an adenovirus-5 vector (Ad-5). The production of ad-5 PacI-linearized plasmids (6 µg) containing the adenovirus genomes, as well as TNMD shRNA, or null sequences were transfected into 1 × 10^6^ HEK293 cells and the viruses were recovered 8–10 days post-transfection. Next, the viruses were sequentially amplified until the infection of 4 × 10^8^ HEK293 cells. Viruses were purified via two consecutive rounds of CsCl isopycnic density ultracentrifugation and desalted using a Sephadex PD-10 column (Amersham Biosciences, Uppsala, Sweden). The viral particles were measured via absorbance of disrupted virions at 260 nm where one O.D. equals 1 × 10^12^ particles per mL, while infective particles were measured via end-point dilution assay through counting the number of hexon-producing cells in triplicate^82^. The production of the vectors was conducted at Unitat de Producció de Vectors Virals-Cbateg, Barcelona, Spain. For adenovirus-shRNA experiments, human differentiated adipocytes were transfected with an Ad-5 containing shRNA-TNMD or shRNA-scrambled as a control using hexadimethrine bromide according to the manufacturer’s protocol. First, to characterize the toxicity of adenovirus transduction in human adipocytes, we monitored the cellular viability in adipocytes that were exposed to different multiplicities of infection (MOI) (0, 10, 50, 100, 300, 500 and 1000 for 48 h) using a Neubauer chamber and trypan blue (4%). No toxicity was observed for the tested range of adenovirus. Subsequently, based on *TNMD* gene inhibition (approximately 90%), the MOI selected was 300 in all subsequent experiments. Forty-eight hours after transfection, the cells were collected.

### RNA isolation and qRT-PCR

Total RNA was extracted from cells using the PeqGOLD HP Total RNA kit (Peqlab, Germany). Isolated RNA was treated with Turbo DNase (Ambion, Life Technologies, Carlsbad, CA, USA). We determined the final RNA concentration and quality, according to the 260/280 ratio, using a NanoDrop2000 (NanoDrop Technologies, Winooski, Vermont, USA). Total RNA (500 ng) was transcribed into cDNA using the iScript cDNA Synthesis Kit (Bio-Rad Laboratories, California, USA). Next, we determined the differential gene expression levels of *TNMD* (330001 PHH12206A, Qiagen, Hilden, Germany), peroxisome proliferator-activated receptor gamma (*PPARγ*), leptin (*LEP*), and adiponectin (*ADIPOQ*) during the adipogenic differentiation via qPCR using specific primer sequences (Table S8). The specific primer sequences were designed using Primer3 (http://bioinfo.ut.ee/primer3-0.4.0/). Primers for glucose transporter 4 (*GLUT4*), interleukin 1-beta (*IL1B*), CCAAT/enhancer-binding protein alpha (*CEBPA*), angiopoietin-like 4 (*ANGPTL4*), tumor necrosis factor alpha (*TNF-α*), hormone-sensitive lipase (*HSL*), adipose triglyceride lipase (*ATGL*), perilipin (*PLIN*), and 5′ AMP-activated protein kinase (*AMPK*) were obtained from Bio-Rad Laboratories, California, USA. qPCR was performed using an ABI Prism 7900HT instrument (Applied Biosystems, Foster City, CA, USA) and SYBR Green PCR Master Mix (Applied Biosystems, Foster City, CA, USA). Hypoxanthine-guanine phosphoribosyltransferase-1 (*HPRT1*) was used as a reference gene for the differentiation experiments. Quantification was performed using the Pfaffl method^83^. Compliance with the minimum information for publication of quantitative real-time PCR experiments (MIQE) was made possible using Bio-Rad’s PrimePCR assays. We calculated the statistical validation of the stability of the reference genes in each sample. Bio-Rad recommends using a <0.5 value, which is the most stable expression in the tested samples. The results are expressed as the fold-change calculated.

### Western blot assays

Protein samples from cell lysates that contain 2.5 µg of protein were mixed with 3X SDS-PAGE sample buffer (100 mM Tris-HCl, pH 6.8, 25% SDS, 0.4% bromophenol blue, 10% β-mercaptoethanol and 2% glycerol), separated via SDS-PAGE using a TGX Any kD gel (Bio-Rad Laboratories, California, USA), and transferred to a nitrocellulose membrane (Bio-Rad Laboratories, California, USA). After incubation in blocking buffer [5% nonfat milk and 0.1% Tween 20 in Tris-buffered saline (TBS)], the membranes were probed with one of the following antibodies: anti-TNMD-N14 (SC-49325; 1:200 in 5% nonfat milk), anti-GLUT4 (H61; 1:100 in 5% nonfat milk), and anti-Angptl4 (sc-373762; 1:500 in 5% BSA), which were acquired from Santa Cruz Biotechnology, CA, USA. Anti-adiponectin (AF1065, R&D Systems, Inc, USA; 1:500 in 5% bovine serum albumin, BSA), anti-PPAR-γ (D69; 1:1000 in 5% BSA), anti-phospho-C/EBP-α (Ser21) (1:1000 in 5% BSA), anti-total AMPK-α, anti-phosphorylated AMPK-α (phospho-AMPKα T172) (both 1:1000 in 5% BSA), anti-AKT (C67E7), and anti-phospho-AKT (Ser473, D9E) (1:1000 in 5% BSA), and anti-phospho-NF-kB p65 (Ser536) (1:500 in 5% BSA) were acquired from Cell Signaling Technologies (Beverly, MA, USA). We purchased anti-α-tubulin (internal control, 1:4000 in 5% nonfat milk) from Sigma. Immunoreactive signals were detected via enhanced chemiluminescence (Super-Signal West Dura Chemiluminescent Substrate, 34075, Thermo Fisher Scientific, Carlsbad, CA, USA). The membrane images were digitally captured and the densitometric analyses were conducted using the ImageJ software. The results were expressed as the fold-change in expression relative to the control. The graph shows a representative crop blot.

### Immunofluorescence analysis

Human ADSCs were seeded on cover glasses and cultured for 2 days. Subsequently, adipogenic differentiation was performed over 14 days. We washed the adipocytes twice with PBS and fixed them with 4% paraformaldehyde for 30 minutes. Next, we incubated the cells with a permeabilization solution (0.5% saponin) for 10 minutes and washed them twice with PBS. Subsequently, the cells were incubated with working buffer (WB) containing 0.05% saponin and 1% bovine serum albumin, for 1 hour. The primary antibodies were anti-GLUT4 (1:100 in WB, H-61) and anti-TNMD-N-14 (1:50 in WB, SC-49325). We incubated the samples at 4 °C overnight and washed the cover glasses three times with a working buffer for 5 min per wash. Next, the secondary antibodies were added, and GLUT4 and TNMD were visualized using an Alexa 488-conjugated chicken anti-goat IgG and Alexa 594-conjugated chicken anti-rabbit IgG at 1:1000 dilutions (Molecular Probes, Thermo Fisher Scientific, Carlsbad, CA, USA). Finally, we used ProLong Gold Antifade Mountant with DAPI (P36931, Molecular Probes, Thermo Fisher Scientific, Carlsbad, CA, USA) to fix cells with cover slips (Menzel-Glaser, 24 × 60 mm #1, Denmark). Image acquisition was performed with cells examined under a Nikon A1 confocal microscope equipped with a 20X immersion objective. Z-series optical sections were collected using a 1-micron-step-size and displayed as maximum z-projections using the NIS Elements/ImageJ software. Image acquisition was additionally performed using a fluorescence microscope (Olympus IX2).

### Intracellular IL-1β and TNF-α protein levels

The intracellular IL-1β and TNF-α levels were determined in cell lysates in the shRNA-TNMD- and shRNA-control-treated adipocytes. Samples were harvested with protein lysis buffer, diluted in the appropriate buffer diluents and added to the wells with the rest of the reagents. IL-1β and TNF-α were determined using a MILLI*plex*^TM^ kit (HSTCMAG-28SK) on a Luminex 200 system (Luminex Corporation, Austin, Tex., USA).

### Glucose-uptake assays

Glucose uptake was determined using a colorimetric assay kit (MAK083, Sigma-Aldrich, St. Louis, MO, USA). Briefly, we differentiated ADSCs in 12-well plates, as described in the “Cell culture and incubation” section. After adenovirus transfection at day 14, we washed the differentiated adipocytes twice with PBS and starved them overnight in a serum-free medium. Next, we washed the cells 3 times with PBS and glucose starved them by incubating for 40 min in KRPH buffer (5 mM Na_2_HPO_4_, 20 mM HEPES, pH 7.4, 1 mM MgSO_4_, 1 mM CaCl_2_, 137 mM NaCl and 4.7 mM KCl) containing 2% BSA. Glucose uptake was assessed with 1 mM 2-deoxy-D-glucose in KRPH for 20 min at 37 °C and 5% CO_2_. As a positive control, the cells were stimulated with insulin (1 μΜ) for 20 min. Glucose uptake levels were expressed in pmol/well.

### Statistical analysis

The results in the tables are presented as the mean (SD). The one-way ANOVA test and Tukey post hoc test were performed to compare phenotype data between obese, overweight, and normal-BMI children. P values < 0.05 were considered statistically significant. These statistical analyses were conducted in R environment^84^. A specific genetic analysis design was implemented in PLINK v1.07^14^ and R environment to handle the X-chromosomal location of *TNMD*; the respective codes are available upon request.

*In vitro* experiments were repeated at least three times. In each experiment, two replicates were performed. Data are expressed as the mean ± standard error of the mean (SEM). Significant differences in the levels of gene and protein expression and glucose uptake were determined using the nonparametric Mann-Whitney *U* test; statistical significance was defined as *P-value < 0.05, **P-value < 0.01. Statistical analyses were performed using SPSS version 22, for Windows (SPSS, Chicago, IL, USA).

## Supplementary information

Supplementary information

## Data Availability

The authors confirm that all data underlying the findings are fully available without restriction. All raw data underlying the findings described in the manuscript are freely available in Figshare, 10.6084/m9.figshare.5258980.
