# Supplementary material for: Effects of X-chromosome Tenomodulin Genetic Variants on Obesity in a Children’s Cohort and Implications of the Gene in Adipocyte Metabolism
Source: Sci Rep. 2019 Mar 8;9:3979. doi: 10.1038/s41598-019-40482-0 (PMC6408551; doi:10.1038/s41598-019-40482-0)
Supplement: Supplementary file 1 — Supplementary information [file 41598_2019_40482_MOESM1_ESM.pdf]

Supplementary information to:

Effects of X-chromosome Tenomodulin Genetic Variants on Obesity in a Children's Cohort and Implications of the Gene in Adipocyte Metabolism

Francisco Javier Ruiz-Ojeda[1,2,* #], Augusto Anguita-Ruiz[1,2*], Azahara I. Rupérez[1], Carolina Gomez-Llorente[1,2,3], Josune Olza[1,2,3], Rocío Vázquez-Cobela[4], Mercedes Gil-Campos[3,6], Gloria Bueno[3,5], Rosaura Leis[3,4], Ramón Cañete[3,6], Luis A. Moreno[5], Angel Gil[1,2,3], Concepcion Maria Aguilera[1,2,3#]

[1]Department of Biochemistry and Molecular Biology II, Institute of Nutrition and Food Technology "José Mataix", Center of Biomedical Research, University of Granada, Avda. del Conocimiento s/n. 18016 Armilla, Granada, Spain.

[2]Instituto de Investigación Biosanitaria IBS.GRANADA, Complejo Hospitalario Universitario de Granada, Granada 18014, Spain.

[3] CIBEROBN (Physiopathology of Obesity and Nutrition Network CB12/03/30038), Instituto de Salud Carlos III (ISCIII), Madrid 28029, Spain.

[4] Unit of Investigation in Nutrition, Growth and Human Development of Galicia, Pediatric Department (USC). Instituto de Investigación Sanitaria de Santiago de Compostela (IDIS), Complexo Hospitalario Universitario de Santiago, Santiago de Compostela, Spain.

[5] Growth, Exercise, NUtrition and Development (GENUD) Research Group, Universidad de Zaragoza, Zaragoza, Spain; Instituto Agroalimentario de Aragón (IA2), Instituto de Investigación Sanitaria de Aragón (IIS Aragón) and Centro de Investigación Biomédica en Red de Fisiopatología de la Nutrición y la Obesidad (CIBEROBN), Zaragoza, Spain.

[6] Department of Paediatrics, Reina Sofia University Hospital, Institute Maimónides of Biomedicine Investigation of Córdoba (IMIBIC), University of Córdoba, Avda Menéndez Pidal s/n, 14004 Córdoba, Spain.

Table S1. General characteristics, biochemistry, adipokines, and biomarkers of inflammation and CVD risk of the children in the study.

	**Normal-weight**	**Overweight**	**Obesity**	
	**n=258**	**n=177**	**n=480**	**p**
**Demography**				
Age (y)	9.77 (2.49) [a]	10.41 (2.52) [b]	10.00 (2.63) [ab]	**0.038**
Sex (f/m)	122/136	109/68	246/234	
**Anthropometry**				
BMI (kg/m^2)	16.91 (2.08) [a]	22.32 (2.68) [b]	27.94 (4.03) [c]	< **0.001**
BMI z-score	-0.28 (0.54) [a]	1.32 (0.48) [b]	3.39 (1.35) [c]	< **0.001**
Waist circumference (cm)	60.18 (7.85) [a]	73.93 (10.99) [b]	88.10 (13.00) [c]	< **0.001**
Waist-to-height ratio	0.44 (0.04) [a]	0.52 (0.06) [b]	0.61 (0.07) [c]	< **0.001**
**Biochemistry**				
Systolic BP (mmHg)	97.92 (12.93) [a]	106.00 (13.95) [b]	111.17 (14.01) [c]	< **0.001**

Diastolic BP (mm Hg)	60.24 (9.27) [a]	63.47 (10.84) [b]	67.49 (11.34) [c]	**< 0.001**
Glucose (mg/dl)	84.26 (7.31)	85.55 (8.02)	84.12 (7.88)	0.102
Insulin (mU/dl)	6.99 (4.49) [a]	10.15 (7.20) [b]	14.25 (9.37) [c]	**< 0.001**
HOMA-IR	1.49 (1.02) [a]	2.18 (1.63) [b]	3.01 (2.13) [c]	**< 0.001**
QUICKI	0.38 (0.04) [a]	0.35 (0.04) [b]	0.34 (0.04) [c]	**< 0.001**
TAG (mg/dl)	54.96 (22.80) [a]	66.72 (31.41) [b]	74.91 (37.03) [c]	**< 0.001**
HDL-c (mg/dl)	64.52 (14.90) [a]	55.89 (14.01) [b]	48.53 (11.95) [c]	**< 0.001**
LDL-c (mg/dl)	90.07 (25.34) [a]	98.42 (29.75) [b]	96.06 (24.69) [b]	**0.002**
oxLDL (mg/dl)	2.13 (2.20)	1.61 (1.53)	1.81 (1.82)	0.117
Apo B (mg/dl)	64.31 (16.10)	67.83 (19.79)	67.32 (18.51)	0.154
Apo A (mg/dl)	153.68 (30.81) [a]	137.79 (30.13) [b]	128.95 (27.91) [c]	**< 0.001**
**Adipokines**				
Adiponectin (mg/l)	21.69 (12.00) [a]	19.20 (11.57) [b]	14.40 (8.78) [c]	**< 0.001**
Leptin (μg/l)	4.17 (4.30) [a]	11.25 (6.48) [b]	21.71 (14.33) [c]	**< 0.001**
ALR	0.28 (0.59) [a]	0.90 (0.85) [b]	1.78 (1.94) [c]	**< 0.001**
Resistin (μg/l)	16.73 (13.80)	15.74 (11.45)	14.95 (10.38)	0.139
**Inflammation**				
hsCRP (mg/l)	0.81 (1.77) [a]	1.49 (2.10) [a]	3.67 (4.71) [b]	**< 0.001**
TNF-α (ng/l)	2.75 (1.61) [a]	2.95 (1.94) [a]	4.90 (3.17) [b]	**< 0.001**

IL-6 (ng/l)	2.83 (5.25)	2.53 (4.51)	3.59 (6.14)	0.074
IL-8 (ng/l)	1.72 (1.75) [a]	1.89 (1.80) [a]	2.64 (2.63) [b]	**< 0.001**
**CVD risk**				
Total PAI-1 (µg/l)	17.44 (12.79) [a]	22.41 (14.43) [b]	27.18 (16.77) [c]	**< 0.001**
MPO (µg/l)	28.65 (43.17) [a]	25.87 (36.73) [a]	48.89 (63.13) [b]	**< 0.001**
sICAM (mg/l)	0.13 (0.07)	0.14 (0.09)	0.14 (0.09)	0.242
sVCAM (mg/l)	1.14 (0.33) [a]	1.08 (0.34) [a]	0.92 (0.37) [b]	**< 0.001**
MMP-9 (µg/l)	91.40 (59.08) [ab]	77.10 (56.06) [a]	112.18 (111.30) [b]	**0.002**

BMI, body mass index; BP, blood pressure; HOMA-IR, homeostasis model assessment for insulin resistance; QUICKI, quantitative insulin sensitivity check index; TAG, triglycerides; HDL-c, high-density lipoproteins-cholesterol; LDL-c, low-density lipoproteins-cholesterol; oxLDL, oxidized low-density lipoprotein; Apo B, apolipoprotein B; Apo A, apolipoprotein A; ALR, adiponectin leptin ratio; hsCRP, high-sensitivity CRP; TNF-α, tumor necrosis factor alpha; IL, interleukin; PAI-1, plasminogen activator inhibitor-1; MPO, myeloperoxidase; sICAM, soluble intercellular cell adhesion molecule-1; sVCAM, soluble vascular cell adhesion molecule-1; MMP-9, matrix metalloproteinase-9. Childhood obesity was defined according to Cole et al. (2000). Data are expressed as mean (standard deviation). Differences in means between groups were analyzed using the Anova One Way test. Distributions within the same row with unlike superscript letters were significantly different (*p <0.05) according to two different post-hoc parametric analyses.

Supplementary table S1 - General characteristics, biochemistry, adipokines, and biomarkers of inflammation and CVD risk of the children in the study. BMI, body mass index; BP, blood pressure; HOMA-IR, homeostasis model assessment for insulin resistance; QUICKI, quantitative insulin sensitivity check index; TAG,

triglycerides; HDL-c, high-density lipoproteins-cholesterol; LDL-c, low-density lipoproteins-cholesterol; oxLDL, oxidized low-density lipoprotein; Apo B, apolipoprotein B; Apo A, apolipoprotein A; ALR, adiponectin leptin ratio; hsCRP, high-sensitivity CRP; TNF-α, tumor necrosis factor alpha; IL, interleukin; PAI-1, plasminogen activator inhibitor-1; MPO, myeloperoxidase; sICAM, soluble intercellular cell adhesion molecule-1; sVCAM, soluble vascular cell adhesion molecule-1; MMP-9, matrix metalloproteinase-9. Childhood obesity was defined according to Cole et al. (2000). Data are expressed as the mean (standard deviation). Differences in means between groups were analyzed using one-way ANOVA. Distributions within the same row with unlike superscript letters were significantly different ($p < 0.05$) according to two different post hoc parametric analyses.

Table S2. Frequencies and association results of haploblock 2 with BMI z-score.

BMI z-score	Markers					B (95 % CI)	p
	rs2073162	rs2073163	rs4828038	rs1155974	Freq.		
**Girls**							
Haplotype	G	T	C	C	0.53	|	
Ref. Haplotype	A	C	T	T	0.44	0.1 (-0.09, 0.29)	0.309
**Boys**							
Haplotype	G	T	C	C	0.57	|	
Ref. Haplotype	A	C	T	T	0.42	**0.634 (0.21, 1.06)**	**0.004**

BMI, body mass index; B, Beta; CI, confidence interval. *P*-value was obtained under an omnibus haplotype test which performs an H-1 df test comparing an alternate model (each haplotype having a unique effect) versus the null model (no haplotypes having any different effect). The Beta coefficients must be interpreted with respect to Ref. Haplotype.

Supplementary table S2 - Frequencies and association results of haploblock 2 with BMI z-score. BMI, body mass index; B, Beta; CI, confidence interval. P value was obtained under an omnibus haplotype test, which performs an H-1 df test comparing an alternate model (each haplotype having a unique effect) versus the null model (no haplotypes having any different effect). The Beta coefficients must be interpreted with respect to Ref. Haplotype.

Table S3. Association between rs4828038 TNMD and biochemistry, adipokines, and biomarkers of inflammation and CVD risk in children.

Phenotype	Genotypes									
	T/T	$n_{T/T}$	T/C	$n_{T/C}$	C/C	$n_{C/C}$	B (95%CI)	*P*-value	B_{BMI} (95%CI)	p_{BMI}
**LDL-c (mg/dl)**										
Females	97.89 (25.66)	130	92.34 (24.17)	168	96.3 (27.87)	171	0.52 (-2.44, 3.48)	0.731[a]	0.45 (-2.50, 3.41)	0.764[a]
Males	94.86 (29.02)	169	NA	0	93.34 (23.92)	231	1.44 (-3.75, 6.63)	0.587[a]	0.77 (-4.43, 5.98)	0.771[a]
**oxLDL (mg/dl)**										
Females	1.78 (1.88)	68	1.62 (1.56)	56	1.68 (1.63)	64	0.07 (-0.22, 0.36)	0.635[a]	0.08 (-0.21, 0.36)	0.604[a]
Males	2.50 (2.05)	65	NA	0	2.03 (2.19)	96	0.47 (-0.21, 1.14)	0.176[a]	0.63 (-0.04, 1.31)	0.069[a]
**Apo B (mg/dl)**										
Females	67.94 (19.92)	96	67.54 (16.01)	102	66.3 (19.54)	104	0.85 (-1.71, 3.4)	0.517[a]	0.9 (-1.66, 3.46)	0.491[a]
Males	65.54 (18.14)	109	NA	0	65.57 (17.07)	152	0.004 (-4.3, 4.31)	0.998[a]	-0.47 (-4.79, 3.86)	0.833[a]
**Apo A (mg/dl)**										
Females	132.5 (33.13)	115	136.8 (27.94)	134	137.4 (31.95)	140	-2.48 (-6.27, 1.30)	0.199[a]	-1.97 (-5.52, 1.57)	0.276[a]
Males	140.7 (29.55)	149	NA	0	141.5 (33.52)	189	-0.75 (-7.6, 6.1)	0.831[a]	2.53 (-4.04, 9.10)	0.450[a]
**hsCRP (mg/l)**										
Females	2.34 (4.52)	95	2.87 (3.74)	126	1.72 (2.22)	118	0.33 (-0.14, 0.81)	0.172[a]	0.22 (-0.23, 0.66)	0.344[a]
Males	2.19 (4.18)	120	NA	0	1.87 (3.31)	160	0.31 (-0.57, 1.19)	0.496[a]	0.02 (-0.81, 0.85)	0.966[a]
**TNF-α (ng/l)**										
Females	3.81 (2.63)	132	3.39 (2.39)	170	4.05 (3)	173	-0.15 (-0.46, 0.15)	0.334[a]	-0.15 (-0.45, 0.14)	0.310[a]
Males	4.27 (3.12)	170	NA	0	3.91 (2.73)	233	0.36 (-0.22, 0.93)	0.222[a]	0.15 (-0.40, 0.70)	0.591[a]
**IL-8 (ng/l)**										
Females	1.95 (1.55)	129	1.99 (1.69)	167	2.35 (2.89)	169	-0.2 (-0.45, 0.05)	0.111[a]	-0.20 (-0.45, 0.04)	0.107[a]

Males	2.35 (2.52)	166	NA	0	2.25 (2.08)	227	0.1 (-0.36, 0.56)	0.667[a]	0.0005 (-0.45, 0.45)	0.998[a]
**Total PAI-1 (µg/l)**										
Females	22.41 (16.46)	109	26.43 (17.95)	151	22.87 (14.68)	144	-0.03 (-2.08, 2.02)	0.975[a]	-0.47 (-2.42, 1.47)	0.633[a]
Males	21.95 (15.12)	138	NA	0	21.48 (14.3)	196	0.57 (-2.6, 3.75)	0.724[a]	-0.19 (-3.29, 2.91)	0.905[a]
**MPO (µg/l)**										
Females	35.52 (42.29)	128	38.89 (55.2)	166	39.85 (58.9)	167	-1.98 (-8.08, 4.11)	0.524[a]	-3.11 (-9.19, 2.97)	0.316[a]
Males	38.21 (45.3)	163	NA	0	41.49 (64.23)	221	-3.33 (-14.9, 8.23)	0.572[a]	-5.17 (-16.79, 6.44)	0.383[a]
**sICAM (mg/l)**										
Females	0.15 (0.13)	129	0.14 (0.07)	170	0.14 (0.09)	171	0.007 (-0.004, 0.02)	0.224[a]	0.007 (-0.004, 0.017)	0.228[a]
Males	0.13 (0.06)	170	NA	0	0.14 (0.08)	233	-0.008 (-0.02, 0.006)	0.252[a]	-0.009 (-0.02, 0.005)	0.227[a]
**sVCAM (mg/l)**										
Females	1.05 (0.37)	92	1.00 (0.38)	91	0.96 (0.38)	98	0.05 (-0.001, 0.1)	0.057[a]	0.04 (-0.004, 0.09)	0.076[a]
Males	1.01 (0.36)	101	NA	0	1.05 (0.38)	139	-0.03 (-0.12, 0.06)	0.484[a]	-0.01 (-0.10, 0.08)	0.831[a]
**MMP-9 (µg/l)**										
Females	91.89 (66.93)	73	87.22 (63.00)	72	78.5 (45.51)	73	7.88 (-1.53, 17.28)	0.102[a]	7.58 (-1.85, 17.02)	0.116[a]
Males	97.06 (116.3)	74	NA	0	112.4 (101.8)	112	-15.59 (-47.32, 16.13)	0.337[a]	-19.4 (-51.38, 12.58)	0.236[a]
**Adiponectin (mg/l)**										
Females	17.6 (10.37)	130	16.74 (9.25)	169	17.69 (11.63)	171	-0.13 (-1.32, 1.05)	0.827[a]	0.11 (-1.04, 1.26)	0.853[a]
Males	17.56 (11.56)	166	NA	0	16.52 (10.78)	224	1.19 (-0.97, 3.35)	0.281[a]	1.77 (-0.36, 3.90)	0.104[a]
**Leptin (µg/l)**										
Females	15.3 (12.19)	128	17.23 (15.5)	169	15 (11.56)	168	0.31 (-1.19, 1.81)	0.683[a]	0.32 (-0.88, 1.54)	0.592[a]
Males	15.05 (15)	163	NA	0	12.8 (12.96)	225	2.26 (-0.53, 5.04)	0.113[a]	0.43 (-1.90, 2.76)	0.717[a]
**ALR**										
Females	1.12 (1.25)	127	1.26 (1.45)	167	1.26 (1.65)	166	-0.06 (-0.23, 0.11)	0.494[a]	-0.10 (-0.25, 0.05)	0.212[a]

Males	1.32 (2.10)	163	NA	0	1.09 (1.58)	221	0.22 (-0.15, 0.58)	0.248[a]	-0.001 (-0.34, 0.33)	0.993[a]
**Resistin (µg/l)**										
Females	14.43 (9.83)	132	16.8 (11.22)	171	16.53 (13.00)	172	-0.95 (-2.24, 0.34)	0.150[a]	-0.95 (-2.24, 0.34)	0.151[a]
Males	15.64 (10.99)	171	NA	0	15.57 (12.75)	234	0.1 (-2.26, 2.48)	0.928[a]	0.38 (-2.00, 2.75)	0.757[a]

LDL-C, low-density lipoprotein cholesterol; oxLDL, oxidized low-density lipoprotein; ApoB, apolipoprotein B; Apo A, apolipoprotein A; hsCRP, high-sensitivity C-reactive protein; TNF-α, tumor necrosis factor alpha; IL, interleukin; PAI-1, plasminogen activator inhibitor-1; MPO, myeloperoxidase; sICAM, soluble intercellular cell adhesion molecule-1; sVCAM, soluble vascular cell adhesion molecule-1; MMP-9, matrix metalloproteinase-9; ALR, adiponectin leptin ratio; MAF, minor allele frequency; β_{BMI}, Beta obtained under an additive model adjusted for BMI; p_{BMI}, p obtained under an additive model adjusted for BMI; CI, confidence interval; NA, not applicable. Data are expressed as mean (standard deviation). Linear regression analyses stratified by sex were performed under an additive model assuming *TNMD* locus escapes from the X-chromosome inactivation process. That is, while the female genotypes were coded 0, 1, or 2, according to 0, 1, or 2 TNMD SNP alleles, the genotypes for males were coded 0 or 1 according to 0 or 1 alleles. [a] Adjusted for age.

Table S4. Allele frequencies in the whole study population and by sex group.

CHR	SNP	BP	A1	MAF (All)	MAF (Females)	MAF (Males)	A2	P	OR
23	rs11798018	100584572	A	0.2687	0.2669	0.2737	C	0.8381	0.97
23	rs5966709	100589508	T	0.3218	0.3253	0.3148	G	0.7525	1.05
23	rs4828037	100590686	C	0.3424	0.3482	0.3301	T	0.5342	1.08
23	rs2073162	100594019	A	0.4501	0.4577	0.4296	G	0.3706	1.12
23	rs2073163	100594053	C	0.4581	0.4664	0.4362	T	0.3523	1.13
23	rs4828038	100596678	T	0.4475	0.4559	0.4251	C	0.3114	1.13
23	rs1155974	100598283	T	0.4400	0.4443	0.4272	C	0.5907	1.07

P and OR columns correspond to P-values and odd ratios obtained with the Fisher exact test for sex-specific allele frequencies (sex differences in allele frequency per SNP). Abbreviations; CHR, Chromosome; SNP, Single Nucleotide Polymorphism; BP, Base Pair; A1, Minor Allele; MAF, Minor Allele Frequency; A2, Alternative Allele; P, P-value; OR, Odd Ratio.

Supplementary table S4 - Allele frequencies in the whole study population and stratified by sex. P and OR columns correspond to P-values and odds ratios obtained with the Fisher exact test for sex-specific allele frequencies (sex differences in allele frequency per SNP). Abbreviations; CHR, Chromosome; SNP, Single Nucleotide Polymorphism; BP, Base Pair; A1, Minor Allele; MAF, Minor Allele Frequency; A2, Alternative Allele; P, P-value; OR, Odds Ratio.

Table S5. Missing frequency quality control (QC) in our selected markers stratified by sex.

CHR	SNP	MISS FREQ (Males)	MISS FREQ (Females)	MISS FREQ (Males-Females)	P
23	rs11798018	0.112	0.038	0.074	**2.008e-05**
23	rs5966709	0.061	**0.004**	0.057	3.113e-07
23	rs4828037	0.064	**0.015**	0.051	**3.457e-05**
23	rs2073162	0.077	**0.008**	0.069	2.858e-08
23	rs2073163	0.143	0.094	0.049	**0.019**
23	rs4828038	0.073	**0.002**	0.070	4.186e-10
23	rs1155974	0.077	**0.002**	0.075	9.728e-11

P column correspond to differential missingness test between sex groups. Asymptotic p-values were obtained by means of Fisher's exact test. SNPs in bold did pass the QC recommended filters. Abbreviations; CHR, Chromosome; SNP, Single Nucleotide Polymorphism; MISS FREQ, Missing Frequency.

Supplementary table S5 - Missing frequency quality control (QC) check in our selected markers stratified by sex. P column correspond to the differential missingness test between sex groups. Asymptotic p-values were obtained by means of a Fisher's exact test. SNPs in bold did pass the QC recommended filters. Abbreviations; CHR, Chromosome; SNP, Single Nucleotide Polymorphism; MISS FREQ, Missing Frequency.

Table S6. Genotype counts and Hardy-Weinberg test statistics for each SNP in the normal-BMI female group.

CHR	SNP	TEST	A1	GENO	O(HET)	E(HET)	P
23	rs11798018	ALL	A	34/177/249	0,3848	0,3908	0,7215
23	rs11798018	AFF	A	25/133/187	0,3855	0,3898	0,89
23	rs11798018	UNAFF	A	9/44/61	0,386	0,396	0,8135
23	rs5966709	ALL	T	67/175/234	0,3676	0,4385	0,000533
23	rs5966709	AFF	T	55/128/171	0,3616	0,4463	0,0005099
23	rs5966709	UNAFF	T	12/46/63	0,3802	0,4112	0,3842
23	rs4828037	ALL	C	75/178/219	0,3771	0,4535	0,0003579
23	rs4828037	AFF	C	63/129/158	0,3686	0,4632	0,000139
23	rs4828037	UNAFF	C	12/48/61	0,3967	0,418	0,6628
23	rs2073162	ALL	A	132/172/170	0,3629	0,4968	4,587E-009
23	rs2073162	AFF	A	99/128/124	0,3647	0,4975	6,776E-007
23	rs2073162	UNAFF	A	33/43/46	0,3525	0,4943	0,001752
23	rs2073163	ALL	C	129/148/156	0,3418	0,4981	6,918E-011

23	rs2073163	AFF	C	98/112/113	0,3467	0,4989	3,83E-008
23	rs2073163	UNAFF	C	31/35/43	0,3211	0,4939	0,0002413
23	rs4828038	ALL	T	133/171/173	0,3585	0,4965	1,564E-009
23	rs4828038	AFF	T	100/128/127	0,3606	0,4971	2,494E-007
23	rs4828038	UNAFF	T	33/43/46	0,3525	0,4943	0,001752
23	rs1155974	ALL	T	127/172/178	0,3606	0,4943	4,18E-009
23	rs1155974	AFF	T	94/128/132	0,3616	0,4942	4,018E-007
23	rs1155974	UNAFF	T	33/43/46	0,3525	0,4943	0,001752

Each SNP has three entries showing results for either ALL, AFF (overweight and children with obesity) or UNAFF (normal-BMI children only) individuals. Hardy Weinberg analysis was performed with the exact test described and implemented by Wigginton et al (2005). Abbreviations; CHR, Chromosome; SNP, Single Nucleotide Polymorphism; A1, minor allele; GENO, genotype counts; O(HET), observed heterozygosity; E(HET), expected heterozygosity; P, hardy weinberg Pvalue.

Supplementary table S6 - Genotype counts and Hardy-Weinberg test statistics for each SNP in the normal-BMI female group. Each SNP has three entries that show results for either ALL, AFF (overweight and children with obesity) or UNAFF (normal-BMI children only) individuals. Hardy Weinberg analysis was performed with the exact test described and implemented by Wigginton et al [87]. Abbreviations; CHR, Chromosome; SNP, Single Nucleotide Polymorphism; A1, minor allele; GENO,

genotype counts; O(HET), observed heterozygosity; E(HET), expected heterozygosity; P, Hardy Weinberg P value.

Table S7. Association between X chromosome SNPs and HOMA-IR, Glucose and BMI z-score in our dataset.

**HOMA-IR**					
SNP	*N*	*Chi.squared.1.df*	*Chi.squared.2.df*	*P.1df*	*P.2df*
rs11798018	811	0,1090131	1,6807819	0,74127057	0,43154178
rs5966709	849	0,1476293	2,7107734	0,70081125	0,25784756
rs4828037	844	0,3548201	2,9189555	0,55139719	0,23235759
rs2073162	841	5,4833947	6,3467175	**0,01919794**	**0,04186276**
rs2073163	773	4,7811041	5,9353704	**0,02877367**	0,0514222
rs4828038	849	6,0045622	7,241449	**0,01426893**	**0,02676328**
rs1155974	844	4,2225706	6,6837048	**0,03988968**	**0,03537138**
rs2011162	839	0,4870565	0,9158886	0,48524328	0,63258272
**Glucose (mg/dl)**					

SNP	*N*	*Chi.squared.1.df*	*Chi.squared.2.df*	*P.1df*	*P.2df*
rs11798018	844	0,004726781	1,0627903	0,94518736	0,58778436
rs5966709	881	0,551359469	0,5905331	0,45776275	0,74433317
rs4828037	876	1,229148881	1,2570217	0,26757262	0,53338551
rs2073162	873	5,173066411	8,1345486	**0,02293969**	**0,017124**
rs2073163	804	2,80036021	4,0061462	0,09424313	0,13492002
rs4828038	880	4,787514682	6,4270721	**0,02866676**	**0,04021416**
rs1155974	876	3,949849694	4,7416028	**0,04687555**	0,09340584
rs2011162	871	0,92907527	3,5561761	0,33510333	0,16896089
**BMI z-score**					
SNP	*N*	*Chi.squared.1.df*	*Chi.squared.2.df*	*P.1df*	*P.2df*
rs11798018	845	0,9734192	1,156192	0,323828907	0,560965399
rs5966709	881	0,7770086	1,214746	0,378057571	0,544780076
rs4828037	877	0,5135826	0,513737	0,473592085	0,773469912

rs2073162	872	8,6167139	9,591307	**0,003330922**	**0,008265597**
rs2073163	803	7,0909178	8,608634	**0,007747556**	**0,013510108**
rs4828038	877	9,0228777	10,382949	**0,002666213**	**0,005563796**
rs1155974	875	7,7558824	8,697477	**0,005353788**	**0,012923106**
rs2011162	871	3,3162038	5,33059	0,068600061	0,069578828

SNPs in bold showed statistically significant associations with presented phenotypes under Clayton Statistics. This test explicitly accounts for random X-inactivation and allows the inclusion of females and males together, increasing thereby the statistical power. P.1df and Chi.squared.1.df columns corresponds to Clayton S1 statistic results while P.2df and Chi.squared.2.df corresponds to Clayton S2 statistic. Abbreviations; SNP, Single Nucleotide Polymorphism; N, number of included subjects in the analysis; HOMA-IR, homeostasis model assessment for insulin resistance; BMI ZSCORE, body mass index adjusted by sex and age.

Supplementary table S7 – Clayton's approach: association between X chromosome SNPs and HOMA-IR, Glucose and BMI z-score in our population. SNPs with P values in bold showed statistically significant associations with presented phenotypes under Clayton Statistics. This test explicitly accounts for random X-inactivation and allows the inclusion of females and males together, thereby, increasing the statistical power. P.1df and Chi.squared.1.df columns correspond to the Clayton S1 statistic results, while P.2df and Chi.squared.2.df corresponds to the Clayton S2 statistic. Abbreviations; SNP, Single Nucleotide Polymorphism; N, number of included subjects in the analysis; HOMA-IR, homeostasis model assessment for insulin resistance; BMI ZSCORE, body mass index adjusted by sex and age.

Table S8. Forward and reverse primer sequences used in the qPCR assays.

Gene	Primer sequence	
	Forward	Reverse
HSL	5'CTTCTGGAAAGCCTTCTGGAACATCACCGA-3'	5'-CTGAGCTCCTCACTGTCCTGTCCTTCAC-3'
ATGL	5'-GACGAGCTCATCCAGGCCAATGTCTG-3'	5'-GATGGTGTTCTTAAGCTCATAGAGTGGCAGG-3'
PPARγ	5'-CTCGAGGACACCGGAGAGG-3'	5'-CACGGAGCTGATCCCAAAGT-3'
PLIN	5'-CTCTCGATACACCGTGCAGA-3'	5'-TGGTCCTCATGATCCTCCTC-3'
HPRT1	5'GAGATGGGAGGCCATCACATTGTAGCCCTC-3'	5'CTCCACCAATTACTTTTATGTCCCCTGTTGACTGGTC3'
LEP	5'-GTTGCAAGGCCCAAGAAGCCCA-3'	5'-CAGTGTCTGGTCCATCTTGGATAAGGTCAGG-3'
ADIPOQ	5'-GGCCGTGATGGCAGAGAT-3'	5'-CCTTCAGCCCGGGTACT-3'

Abbreviations: HSL, hormone sensitive lipase; ATGL, adipose triglyceride lipase; PPARγ, peroxisome proliferator-activated receptor gamma; PLIN, perilipin; HPRT1, Hypoxanthine-guanine phosphoribosyltransferase-1; LEP, leptin; ADIPOQ, adiponectin.

Supplementary figure S1

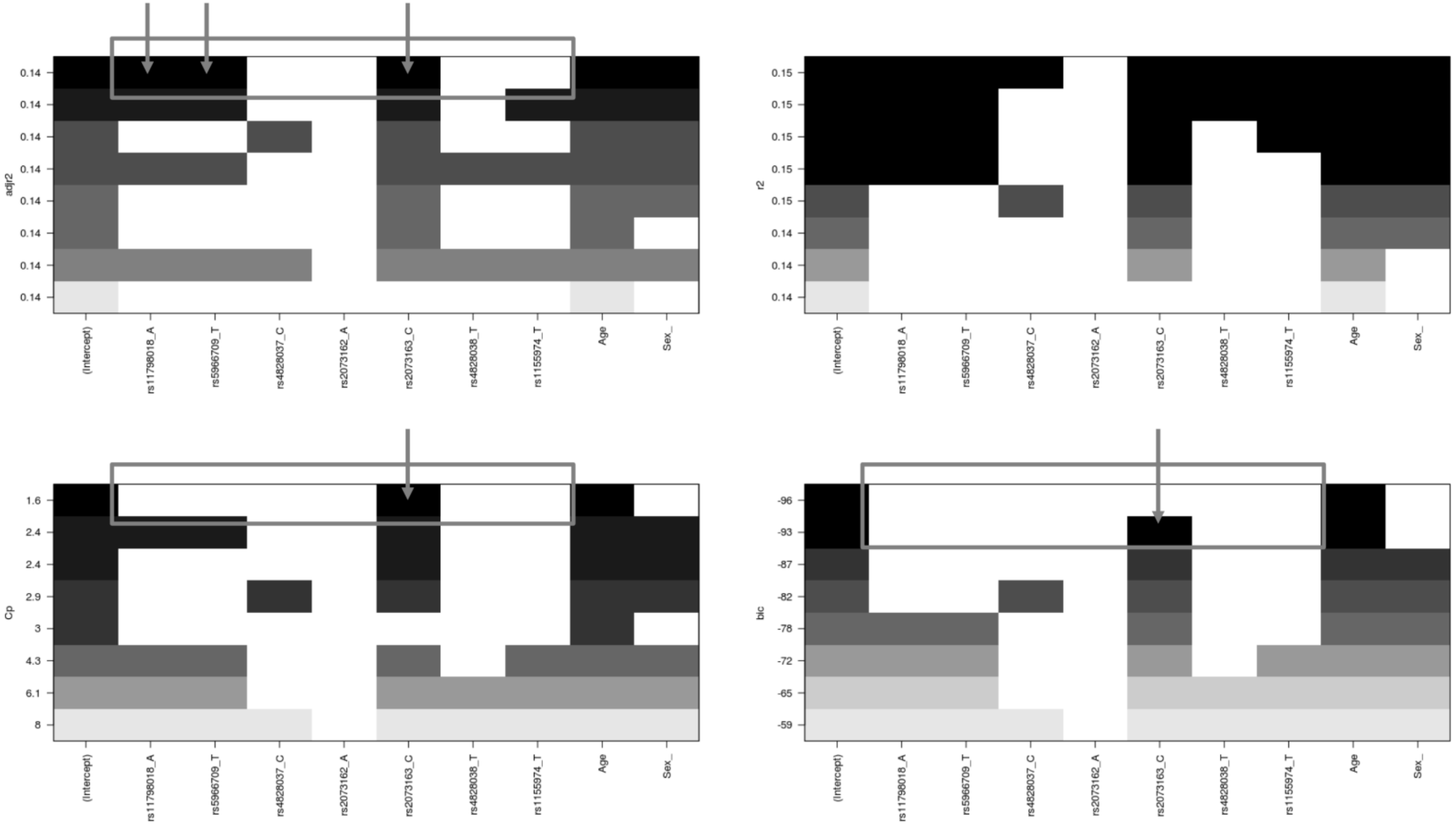

Supplementary figure S1 - Backward stepwise selection on the BMI z-score outcome. Backward selection procedure was employed to identify independent associated SNPs iteratively over all tested SNPs and predictors. Three control parameters were evaluated to select the final models; Cp (which, for the least squares models, is equal to the AIC), the adjusted R^2 and the Bayesian information criterion (BIC). Framed in a rectangle can be found the best-selected models by the control parameter. Marked with arrows, we show the independent signals affecting the outcome.

Supplementary figure S2

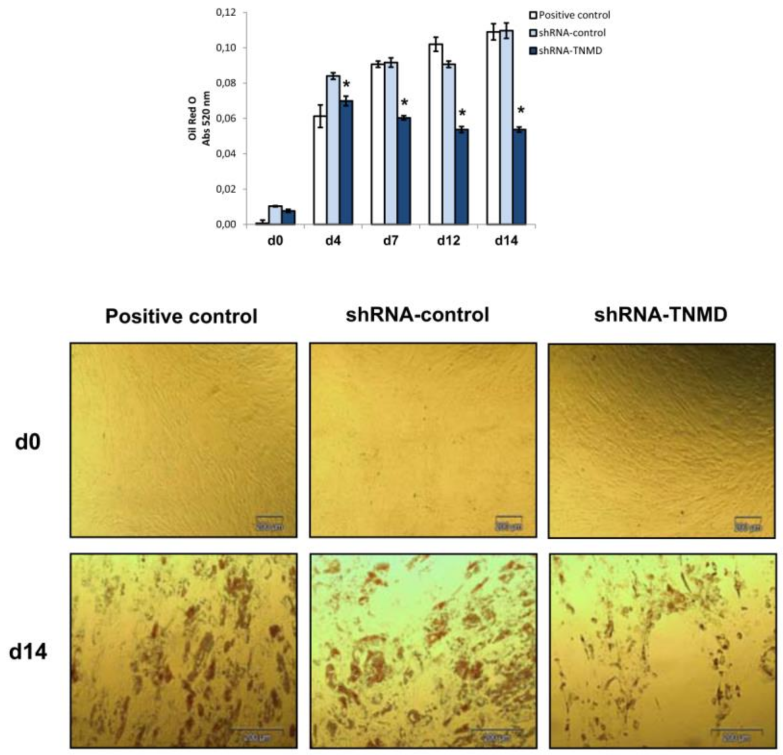

Supplementary figure S2 - shRNA-mediated silencing of *TNMD* impairs adipogenesis in ADSCs. Human adipocytes were transfected with an adenovirus-5 containing a shRNA-TNMD and shRNA-control (scrambled) 2 days before adipogenesis induction. Oil Red O staining was performed on the stated days of differentiation. (a) Optical microscopy images from Oil-Red O staining of ADSCs at day 0 (d0) and day 14 (d14). (b) Lipid content was measured via quantification of

Oil red O staining (absorbance at 495 nm). All values are expressed as the means ± SEM of three independent experiments. Significant differences were identified using the nonparametric Mann-Whitney U test; *p <0.05.

Supplementary figure S3

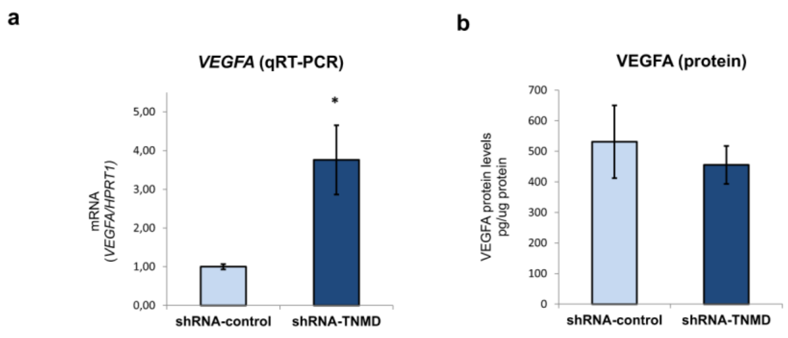

Supplementary figure S3 - Vascular endothelial growth factor A (VEGFA) gene and protein expression when *TNMD* was silenced**.** Human adipocytes were transfected with an adenovirus-5 containing a shRNA-TNMD and shRNA-control (scrambled) at day 14 of adipogenesis induction. **(a)** *VEGFA* mRNA levels were normalized to those of hypoxanthine-guanine phosphoribosyltransferase-1 (*HPRT1*), and the data from three independent experiments are presented as the fold-change, which was calculated using the Pfaffl method. **(b)** VEGFA protein levels were analyzed using the XMap technology (Luminex) as indicated in the methods section. The data from three independent experiments are presented as the means ± SEM. Significant differences were identified using the Mann-Whitney U test; *p <0.05.

Supplementary figure S4

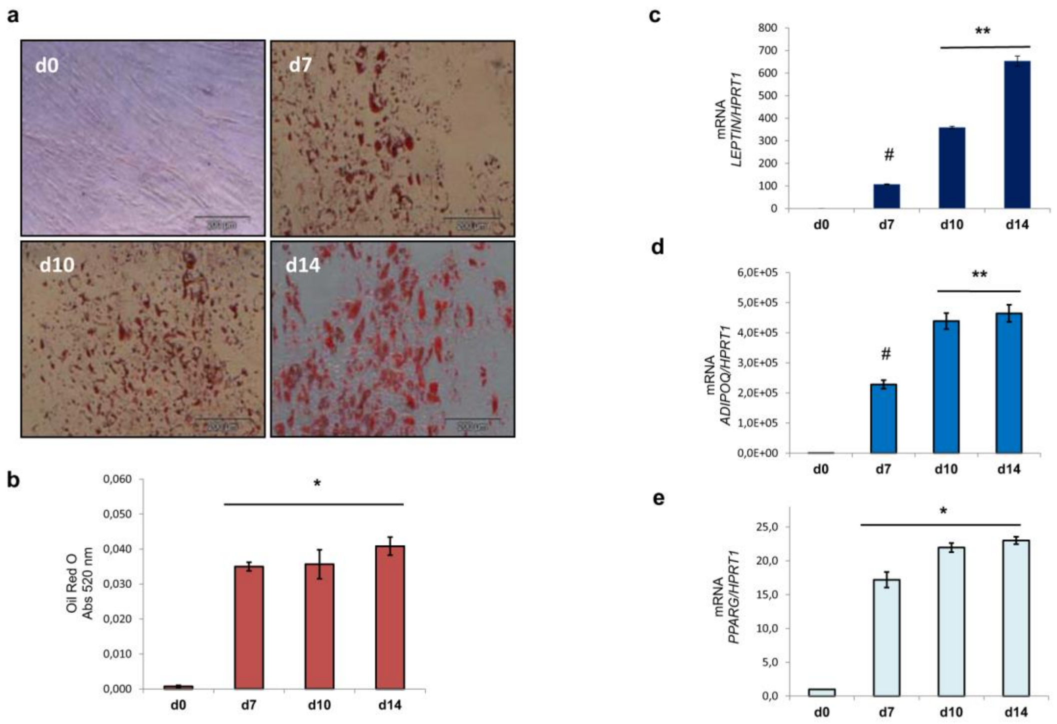

Supplementary figure S4- Adipogenic characterization of human adipose-derived stem cells (ADSCs). (a) Optical microscopy images from Oil-Red O staining of ADSCs at day 0 (d0) and during the adipogenic differentiation at days 7, 10, and 14 (d7, d10, and d14). (b) Lipid content was measured through quantification of oil red-O staining (absorbance at 495 nm). (c, d, e) Leptin (*LEP*), adiponectin (*ADIPOQ*), and peroxisome proliferator-activated receptor gamma (*PPARG*) expression

during adipogenic differentiation. The *LEP*, *PPARG*, and *ADIPOQ* mRNA levels were normalized to those of hypoxanthine-guanine phosphoribosyltransferase-1 (*HPRT1*). The data from three independent experiments are presented as the fold-change, which was calculated using the Pfaffl method. The white bars represent the ADSCs at day 0 (d0) and during the adipogenic differentiation on d7, d10, and d14. All values are expressed as the means ± SEM of three independent experiments. Significant differences were analyzed using the Mann-Whitney U test; d0 vs d10 and d14 *p <0.05; d0 vs d7 #p <0.05.; d0 vs d10 and d14 **p <0.01.

Supplementary figure S5

Supplementary Figure S5

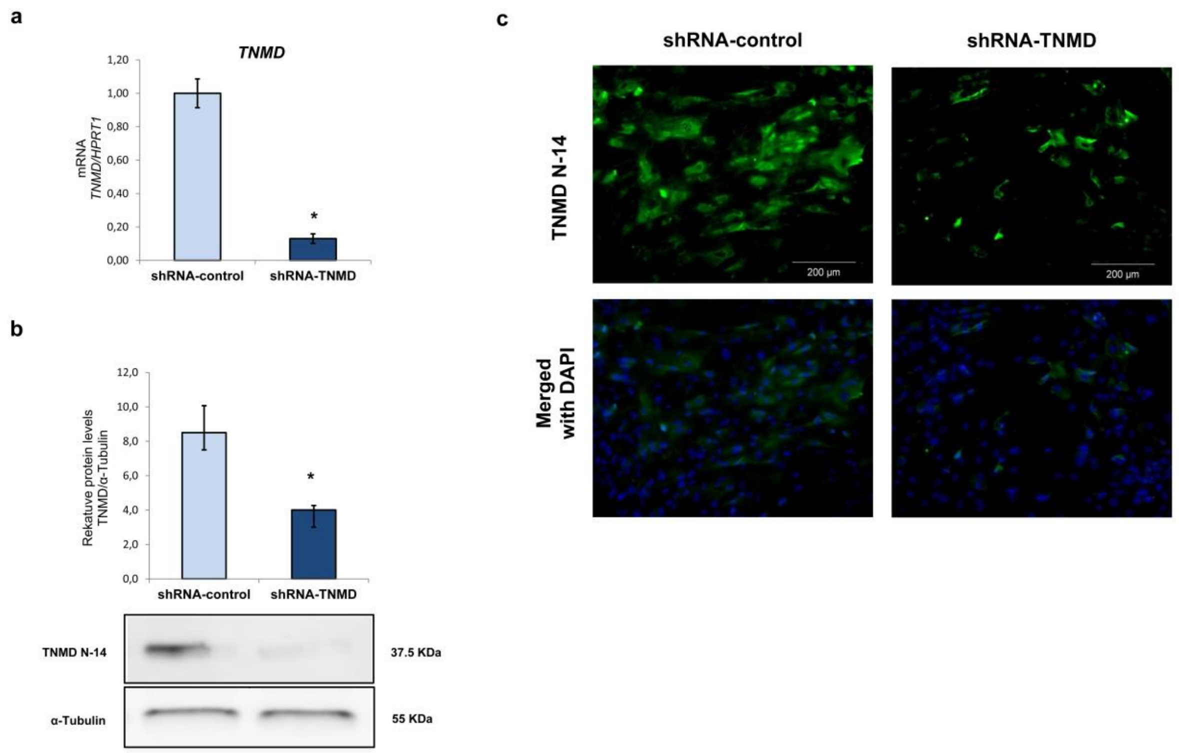

Supplementary figure S5 - shRNA-mediated silencing of *TNMD* gene in human differentiated adipocytes. Human adipocytes were transfected with an adenovirus-5 containing a shRNA-TNMD and shRNA-control (scrambled) at day 14 of adipogenesis induction. (a) *TNMD* mRNA levels were normalized to those of hypoxanthine-guanine phosphoribosyltransferase-1 (*HPRT1*), and the data from three independent experiments are presented as the fold-change, which was calculated using the Pfaffl

method. (b) TNMD protein levels from cell lysates, as analyzed via Western blotting using a specific antibody against TNMD (N-14), normalized to the internal control (α-tubulin), and expressed as fold-change. (c) Immunofluorescent staining of adipocytes at day 14 with N-14 terminal domain of TNMD (green) and 4.6-diamidino-2-phenylindole (DAPI; blue; scale bar, 200 μm) in the shRNA-control and shRNA-TNMD-treated adipocytes. All values are expressed as the means ± SEM of three independent experiments. Significant differences were identified using the nonparametric Mann-Whitney U test; *$p < 0.05$.

Supplementary Methods and Results

Adipogenic characterization of ADSCs

Firstly, adipogenic was monitored via morphological examination of the cellular accumulation of lipid droplets by staining with Oil Red O (Supplementary Figure S4A). In addition, we determined the gene expression of *LEP*, *ADIPOQ*, and *PPARG*, which are the main adipogenic markers, by RT-qPCR (Supplementary Figure S4B). In agreement with previous results [1,2], we observed an increase of *LEP*, *ADIPOQ* and *PPARG* expression from day 0 to day 14.

Inhibition of TNMD in human adipocytes

Human adipocytes at day 14 were transfected with an adenovirus-5 containing shRNA-TNMD or shRNA-control (scrambled). Gene expression showed that

TNMD was down-regulated significantly in the shRNA-TNMD-treated adipocytes compared with the control. Western blot analyses showed that TNMD was lower in the shRNA-TNMD-treated adipocytes by using the anti-TNMD-N-14 terminal domain (Supplementary Figure S5). In addition, immunofluorescence showed protein levels of TNMD, and we observed lower protein levels when *TNMD* was silenced (Supplementary Figure S2).

Supplementary references

1. Wu, Z. et al. Cross-Regulation of C / EBP a and PPAR g Controls the Transcriptional Pathway of Adipogenesis and Insulin Sensitivity. Mol. Cell 3, 151–158 (1999).

2. Vater, C., Kasten, P. & Stiehler, M. Culture media for the differentiation of mesenchymal stromal cells. Acta Biomater. 7, 463–477 (2011).

Western blot images

Supplemental Info Supplemental Figure 5S

TNMD

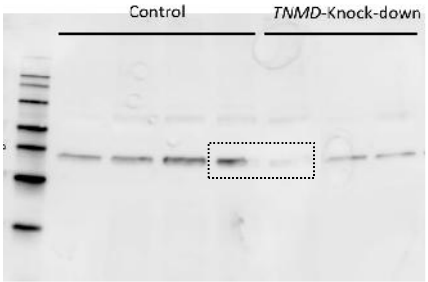

37 KDa

α-Tubulin

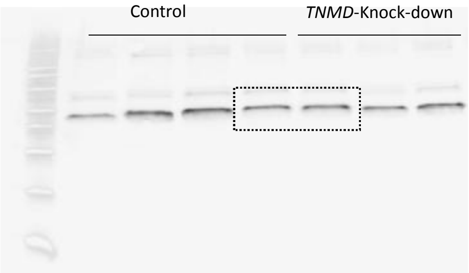

50 KDa

Supplemental Info Figure 2

TNMD

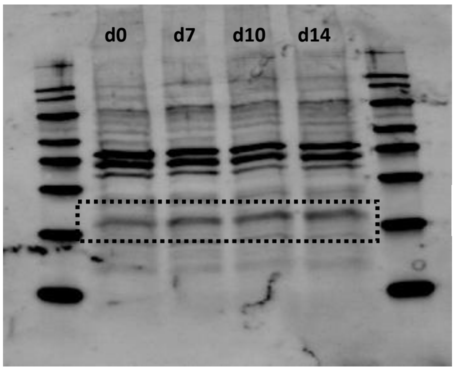

37 KDa

α-Tubulin

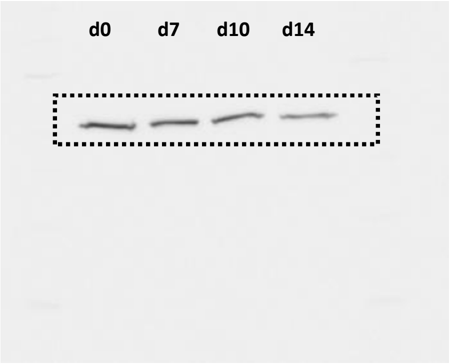

50 KDa

Supplemental Info Figure 3

PPARG

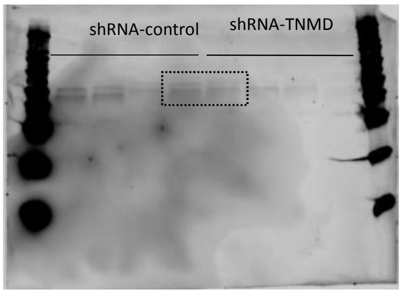

53-57 KDa

α-Tubulin

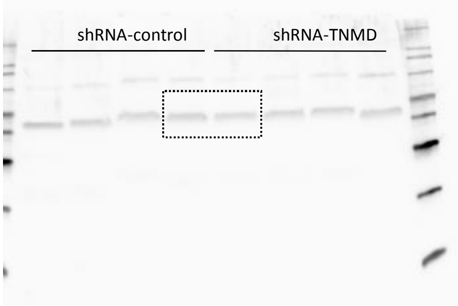

50 KDa

p-C/EBPα

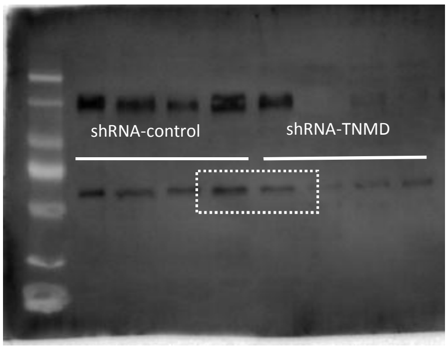

45 KDa

α-Tubulin

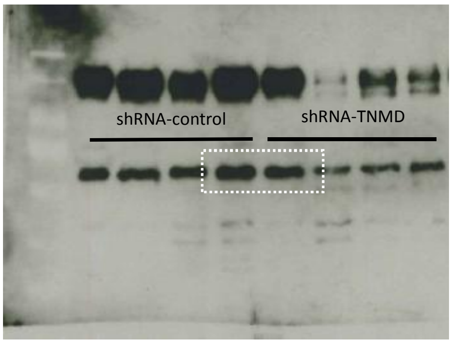

50 KDa

ANGPTL4

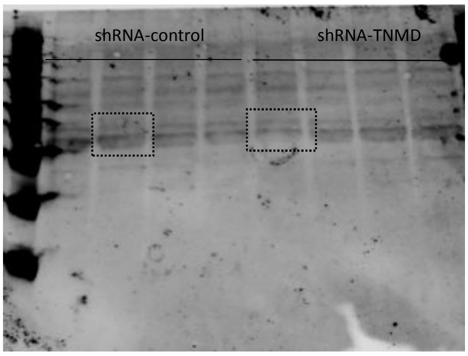

50 KDa

α-Tubulin

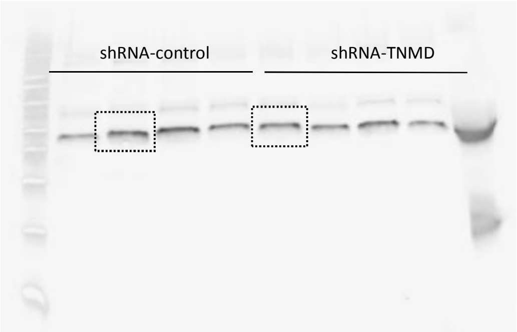

50 KDa

Supplemental Info Figure 4

GLUT4

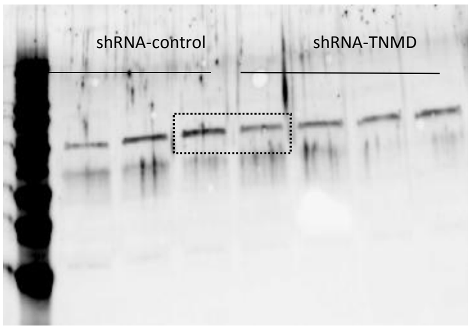

53 KDa

α-Tubulin

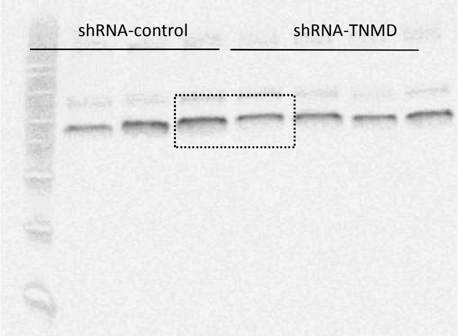

50 KDa

Adiponectin

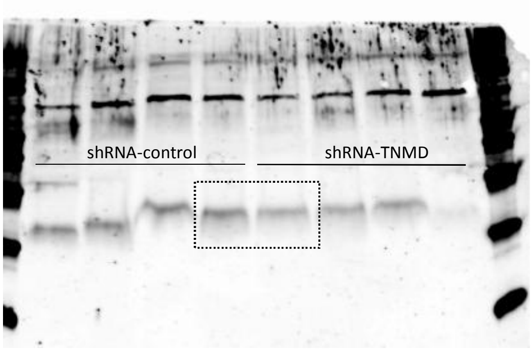

37 KDa

α-Tubulin

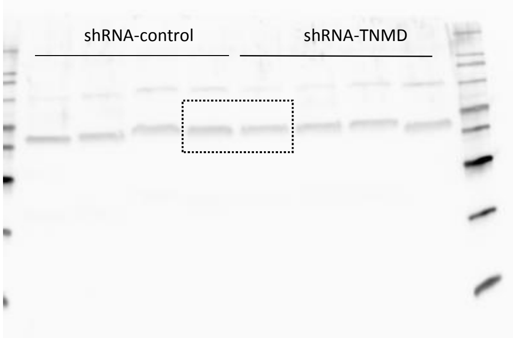

50 KDa

p-AKT (Ser 473)

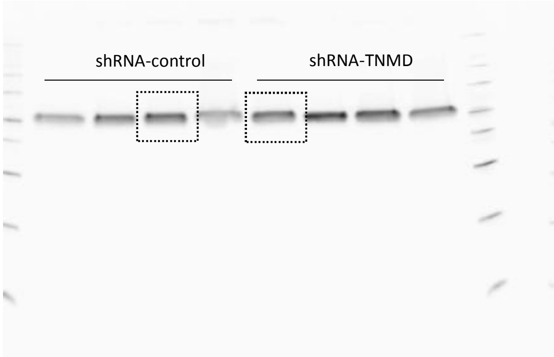

AKT

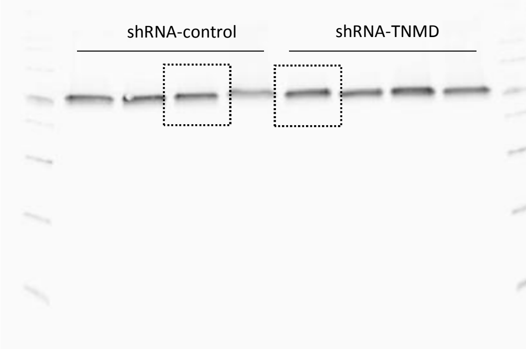

60 KDa

phospho AMPK

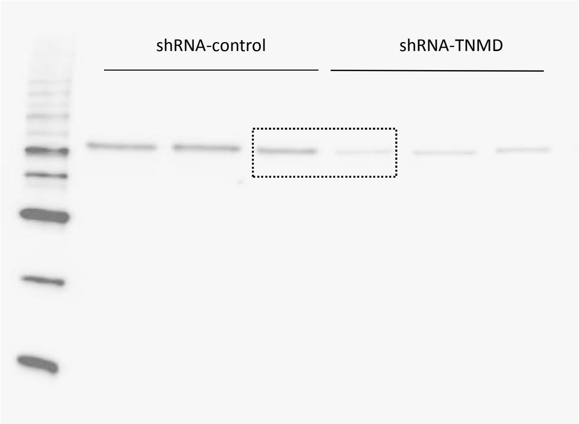

62 KDa

AMPK

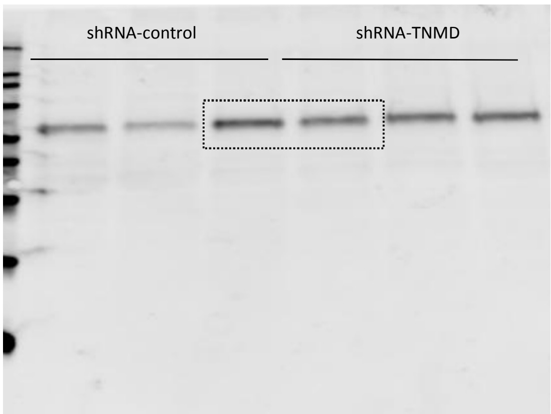

62 KDa

Supplemental Info Figure 5

phospho NFκB p65

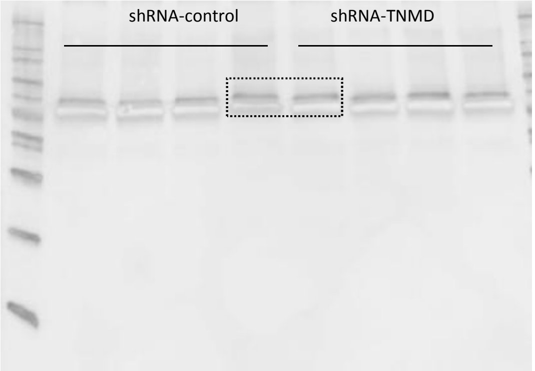

65 KDa

α-Tubulin

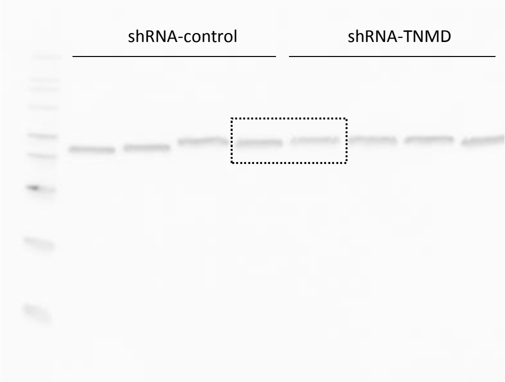

50 KDa
